# A maximum kernel-based association test to detect the pleiotropic genetic effects on multiple phenotypes

**DOI:** 10.1093/bioinformatics/btad291

**Published:** 2023-04-27

**Authors:** Jinjuan Wang, Mingya Long, Qizhai Li

**Affiliations:** School of Mathematics and Statistics, Beijing Institute of Technology, Beijing 100081, China; LSC Academy of Mathematics and Systems Science, Chinese Academy of Sciences, Beijing 100190, China; University of Chinese Academy of Sciences, Beijing 101408, China; LSC Academy of Mathematics and Systems Science, Chinese Academy of Sciences, Beijing 100190, China; University of Chinese Academy of Sciences, Beijing 101408, China

## Abstract

**Motivation:**

Testing the association between multiple phenotypes with a set of genetic variants simultaneously, rather than analyzing one trait at a time, is receiving increasing attention for its high statistical power and easy explanation on pleiotropic effects. The kernel-based association test (KAT), being free of data dimensions and structures, has proven to be a good alternative method for genetic association analysis with multiple phenotypes. However, KAT suffers from substantial power loss when multiple phenotypes have moderate to strong correlations. To handle this issue, we propose a maximum KAT (MaxKAT) and suggest using the generalized extreme value distribution to calculate its statistical significance under the null hypothesis.

**Results:**

We show that MaxKAT reduces computational intensity greatly while maintaining high accuracy. Extensive simulations demonstrate that MaxKAT can properly control type I error rates and obtain remarkably higher power than KAT under most of the considered scenarios. Application to a porcine dataset used in biomedical experiments of human disease further illustrates its practical utility.

**Availability and implementation:**

The R package *MaxKAT* that implements the proposed method is available on Github https://github.com/WangJJ-xrk/MaxKAT.

## 1 Introduction

The advances in high-throughput technology allow researchers to investigate the occurrence, development and treatment of disease from the genetic perspective. Up to now, over ten thousands of disease-associated single-nucleotide polymorphisms (SNPs) have been detected via genome-wide association study (GWAS) based on single trait per variant analysis. However, GWAS suffers from power loss because of weak marginal effects, ignorance of interactions among genetic variants, and strict multiple comparison policy. To make up for these defects, the analysis based on gene sets is a good alternative, where genetic variables are grouped into different sets based on biological knowledge. Many gene-based tests for analysis between a single trait and multiple SNPs have been proposed. For example, Yu et al. (2009) proposed an adaptive rank truncated product test, Wu et al. (2011) developed a sequence kernel association test (SKAT), Pan et al. (2014) constructed an adaptive sum of powered U-score (aSPU) test, and Svishcheva et al. (2019) extended diverse powerful methods developed for gene-based association analysis to situations using the GWAS summary statistics as inputs.

There is growing evidence suggesting the wide existence of pleiotropic genetic effects, i.e. many SNPs are prone to be simultaneously associated with several human complex traits, especially those located in one gene or one pathway (Stearns 2010, Razeto-Barry et al. 2011, Zhang et al. 2017, Wu et al. 2022, Bu et al. 2023). For example, the gene coding for the enzyme phenylalanine hydroxylase not only relates to phenylketonuria, but also affects multiple phenotypes via diverse mutations within this gene, such as mental retardation, seizures, and hair pigmentation (Paul 2000). As a result, it is rational to conduct joint analysis of multiple phenotypic traits. The RV coefficient (Escoufier 1973), which generalizes the Pearson correlation coefficient to multivariate situation, is a useful tool for this issue and provides insight into linear relationships between two sets of variables. And to accommodate diverse association types, it has been extended to cases of nonlinear relationships by using two definite kernel functions instead of the linear kernel, and the corresponding test is known as the kernel-based association test (KAT). KAT has been widely used in genetic and genomic studies. For example, Broadaway et al. (2016) used KAT to test cross-phenotype effects of rare variants, Chen et al. (2016) used the kernel-based regression model to conduct microbiome association studies, Lee et al. (2017) proposed a SKAT-based multivariate association procedure to identify rare variants related to multiple phenotypes, Zhan et al. (2017a) proposed a kernel-based RV statistic to investigate the association between host gene expression and microbiome composition, and Zhan et al. (2017b) generalized the KAT to test associations for high-dimensional structured phenotypes.

Despite its wide usage, KAT is subject to substantial power loss when multiple phenotypes are moderately to strongly correlated. To overcome this drawback, we propose a maximum kernel-based association test (MaxKAT). We first conduct eigenvalue decomposition on KAT and rewrite KAT as a weighted summation of angles between two eigenspaces. By adjusting the weights, we establish a series of tests to describe the association strength at different levels. Since the optimal test is unknown, we choose the most significant one among them as the final test. For convenience of calculation, a generalized extreme value distribution is used to calculate the statistical significance of MaxKAT under the null hypothesis. In addition, the proposed test can accommodate high-dimensional data and yield high power against various alternative hypotheses. Extensive simulation studies show its superior performance over KAT via a wide range of scenarios. And application to a biomedical porcine dataset demonstrates its efficiency in practical situations.

## 2 Materials and methods

Suppose *n* individuals are randomly sampled from a source population. For each individual, data of *m* SNPs in a target gene or region on a chromosome and $\tilde{m}$ phenotypes of interest are collected. Let $X={\left(x_{1},x_{2},\ldots ,x_{n}\right)}^{⊤}$ and $Y={\left(y_{1},y_{2},\ldots ,y_{n}\right)}^{⊤}$ be the corresponding *n *×* m* and $n\times \tilde{m}$ observed data matrix, respectively, where the superscript ${}^{⊤}$ denotes the transpose of a matrix or a vector, $x_{i}={\left(x_{i1},x_{i2},\ldots ,x_{im}\right)}^{⊤}$ and $y_{i}={\left(y_{i1},y_{i2},\ldots ,y_{i\tilde{m}}\right)}^{⊤}$ are the genotypic and phenotypic data for the *i*th individual, respectively, i=1,2,…,n. The null hypothesis holds that these *m* SNPs are not associated with $\tilde{m}$ phenotypes, and the alternative hypothesis is that at least one SNP is associated with at least one phenotype.

### 2.1 Weight-adjusted KATs

Given two definite kernel functions k(·,·) and $\tilde{k}(\cdot ,\cdot )$, two similarity matrices $S={\left(s_{ij}\right)}_{n\times n}$ and $\tilde{S}={\left({\tilde{s}}_{ij}\right)}_{n\times n}$ can be calculated via $s_{ij}=k(x_{i},x_{j})$ and ${\tilde{s}}_{ij}=\tilde{k}(y_{i},y_{j})$, respectively, for i,j=1,2,…,n. Then KAT (Chen et al. 2016, Lee et al. 2017, Zhan et al. 2017a,b) is where tr(·) indicates the trace of a matrix and $H=I_{n}-\frac{1}{n}1_{n}1_{n}^{⊤}$ is the centering matrix with $I_{n}$ being the identity matrix and $1_{n}$ being an *n*-dimensional column vector with all 1 units.


$$\text{KAT}=\frac{1}{n}\text{tr}(HSH\tilde{S}H),$$

To overcome the defect of KAT and develop a powerful and robust test, we take another look at KAT. Based on eigenvalue decomposition strategy, we have where $\lambda_{1}\geq \lambda_{2}\geq \cdots \geq \lambda_{n}$ and ${\tilde{\lambda }}_{1}\geq {\tilde{\lambda }}_{2}\geq \cdots \geq {\tilde{\lambda }}_{n}$ are the eigenvalues of HSH and $H\tilde{S}H$, respectively, and $q_{1},q_{2},\ldots ,q_{n}$ and ${\tilde{q}}_{1},{\tilde{q}}_{2},\ldots ,{\tilde{q}}_{n}$ are their corresponding eigenvectors. Then KAT can be rewritten as


$$HSH=\sum_{i=1}^{n}\lambda_{i}q_{i}q_{i}^{⊤},\;H\tilde{S}H=\sum_{j=1}^{n}{\tilde{\lambda }}_{j}{\tilde{q}}_{j}{\tilde{q}}_{j}^{⊤},$$

$$\text{KAT}=\frac{1}{n}\sum_{i=1}^{n}\sum_{j=1}^{n}\lambda_{i}{\tilde{\lambda }}_{j}{\left(q_{i}^{⊤}{\tilde{q}}_{j}\right)}^{2}.$$

This indicates that KAT is a weighted summation of angles between two sets of eigenvectors. These angles represent the association strengths between genotypes and phenotypes projected on different coordinate levels, and their weights are determined by the corresponding eigenvalues. This summation, however, may cause information loss when some important information contained in angles corresponding to small eigenvalues is concealed owing to their small weights. To deal with this issue, the relative differences among weights need to be adjusted to ensure that important association information becomes visible in the summation. However, the angles vary across different situations and the optimal weights are difficult to know beforehand. Therefore, we next consider power weights and construct a series of weight-adjusted KATs (abbreviated as wKAT) by changing weights in KAT as where *θ* and $\tilde{\theta }$ are two positive constants. Note that the parameter $\frac{1}{n}$ in KAT has been changed to $\frac{1}{n^{\theta +\tilde{\theta }-1}}$ in $\text{wKAT}(\theta ,\tilde{\theta })$ for the convenience of asymptotic property derivation, which does not influence the statistical significance of wKAT.


$$\text{wKAT}(\theta ,\tilde{\theta })=\frac{1}{n^{\theta +\tilde{\theta }-1}}\sum_{i=1}^{n}\sum_{j=1}^{n}\lambda_{i}^{\theta }{\tilde{\lambda }}_{j}^{\tilde{\theta }}{\left(q_{i}^{⊤}{\tilde{q}}_{j}\right)}^{2},$$

Obviously, when $\theta =\tilde{\theta }=1,\;\text{wKAT}(\theta ,\tilde{\theta })$ becomes KAT. It is expected that large weights should give to small angles and small weights to large angles. To detect how *θ* and $\tilde{\theta }$ affect the power of $\text{wKAT}(\theta ,\tilde{\theta })$, we conduct simulations to see their performances. Simulation results show that when elements within multiple variates (such as the *m* dimensional genotypes) are weakly related, index (such as *θ*) larger than 1 helps wKAT obtain higher power than KAT does. On the other hand, if elements within multiple variates (such as the $\tilde{m}$ dimensional phenotypes) are strongly related, index (such as $\tilde{\theta }$) should set to be less than 1 to boost the power. The detailed simulation settings and simulation results are given in Supplementary Section S1 in the Supplementary Materials.

The simulations presented above for wKAT indicate that the distribution of eigenvalues of kernel matrices is affected by the correlation among variables, and the resulting eigenvalue-based weights impact the performance of wKAT. To give a succinct explanation for this phenomenon, we use a linear kernel $k(y_{1},y_{2})=\sum_{r=1}^{\tilde{m}}y_{1r}y_{2r}$ to construct a kernel matrix. Under this condition, the eigenvalues of the kernel matrix are *n*–1 times those of the sample covariance matrix. Therefore, when the correlation strengths among variables are strong, the eigenvalue distribution of the covariance matrix becomes uneven, resulting in an uneven eigenvalue distribution of the kernel matrix. For instance, let us consider $\tilde{m}=100$. When multiple variants are independent with $\Delta_{y}=I_{\tilde{m}}$, all the eigenvalues of Δ_*y*_ are 1. However, when multiple variants are correlated, and $\Delta_{y}=(1-\rho )I_{\tilde{m}}+\rho 1_{\tilde{m}}1_{\tilde{m}}^{⊤}$ with ρ=0.1, the first eigenvalue of Δ_*y*_ is 10.9, and the remaining 99 eigenvalues are all 0.9. Similarly, when $\Delta_{y}=(1-\rho )I_{\tilde{m}}+\rho 1_{\tilde{m}}1_{\tilde{m}}^{⊤}$ with ρ=0.9, the first eigenvalue of Δ_*y*_ is 90.1, with the rest of the eigenvalues being 0.1. Therefore, if variables are strongly related, the eigenvalue distribution of the kernel matrix is very uneven. Under this condition, signals in KAT weighted by small weights are potentially to be masked by those with larger weights, which leads to a loss of power in KAT. Powers of eigenvalues (i.e. $\theta ,\tilde{\theta }$) less than 1 can alleviate this uneven weight distribution, making all the signals visible. Thus, power weight adjustment in wKAT can help enhance its power.

We can write $\text{wKAT}(\theta ,\tilde{\theta })$ as where ${\left(HSH\right)}^{\theta }$ can be seen as an extension of HSH that depicts similarities between pairs of $x_{i}$s at a different level indexed by *θ*. Denote the (*i*, *j*)th element of ${\left(HSH\right)}^{\theta }$ as $\delta_{\theta ,ij}$, and that of HSH as *δ_ij_*. Given θ=0.5, we have $\delta_{ij}=\sum_{l=1}^{n}\delta_{\theta ,il}\delta_{\theta ,jl}$. This indicates that *δ_ij_* calculates similarity between the *i*th and *j*th subjects by integrating their respective similarities with all the other subjects at the level of θ=0.5 as a whole. Similarly, when *θ *= 2, $\delta_{\theta ,ij}=\sum_{l=1}^{n}\delta_{il}\delta_{jl}$, indicating that $\delta_{\theta ,ij}$ calculates similarity between the *i*th and *j*th subjects by integrating their respective similarities with all the other subjects measured by the kernel function k(·,·) as a whole. So compared with *δ_ij_*, $\delta_{\theta ,ij}(\theta \neq 1)$ can be treated as a similarity measure to some extent.


$$\text{wKAT}(\theta ,\tilde{\theta })=\frac{1}{n^{\theta +\tilde{\theta }-1}}\text{tr}({\left(HSH\right)}^{\theta }{\left(H\tilde{S}H\right)}^{\tilde{\theta }}),$$

### 2.2 A maximum kernel-based association test

Although $\text{wKAT}(\theta ,\tilde{\theta })$ is valid for arbitrary positive constants *θ* and $\tilde{\theta }$, good choices on *θ* and $\tilde{\theta }$ can lead to power gains over KAT. Since the optimal choices of *θ* and $\tilde{\theta }$, which are always unknown beforehand, vary with the true relationships between genotypes and phenotypes, we propose a MaxKAT as where Θ and $\tilde{\Theta }$ are two sets of candidate choices for *θ* and $\tilde{\theta }$, respectively, and ${\hat{\mu }}_{\theta \tilde{\theta }}$ and ${\hat{D}}_{\theta \tilde{\theta }}^{2}$ are respective estimates of the mean and variance of $\text{wKAT}(\theta ,\tilde{\theta })$, which can be expressed as


$$\text{MaxKAT}=\underset{\theta \in \Theta ,\tilde{\theta }\in \tilde{\Theta }}{\max}\frac{\text{wKAT}(\theta ,\tilde{\theta })-{\hat{\mu }}_{\theta \tilde{\theta }}}{{\hat{D}}_{\theta \tilde{\theta }}},$$

$${\hat{\mu }}_{\theta \tilde{\theta }}=\frac{1}{n^{\theta +\tilde{\theta }}}\sum_{i,j=1}^{n}\lambda_{i}^{\theta }{\tilde{\lambda }}_{j}^{\tilde{\theta }},\;{\hat{D}}_{\theta \tilde{\theta }}^{2}=\frac{2}{n^{2(\theta +\tilde{\theta })}}\sum_{i,j=1}^{n}\lambda_{i}^{2\theta }{\tilde{\lambda }}_{j}^{2\tilde{\theta }}.$$

We would like to point out that smaller *θ*s (those approaching zero) make all the eigenvalues approach one, resulting in almost the same weights for different angles. In contrast, larger *θ*s may highlight angles corresponding to large eigenvalues overly. In order to strike a balance, candidate values for *θ* and $\tilde{\theta }$ are set to be close to one. The results of simulations and real data studies show that $\Theta =\tilde{\Theta }=(\frac{1}{2},\frac{3}{4},1,2,3)$ is sufficient in most scenarios, and we use this setting in our work.

Since the joint distribution of multiple wKATs is complex, we apply the permutation procedure to the significance calculation for MaxKAT. Once calculating the observed MaxKAT using the original data, we simultaneously permute the rows and columns of matrix $H\tilde{S}H$ for a great many times (denoted as *N*), say 1000 times, and plug the permuted $H\tilde{S}H$ into the expression of MaxKAT to obtain *N* permuted MaxKATs. Then the *P*-value of the observed MaxKAT is the proportion of all these permuted MaxKATs larger than the observed one.

On the other hand, since MaxKAT is the maximum over a Gaussian random field (See Theorem 2 of Supplementary Section S2 in the Supplementary Materials), we can use the generalized extreme value (GEV) distribution to approximate its distribution. The cumulative distribution function of a GEV random variable *Z* is where κ,α,η are unknown parameters. Denote the sample mean, variance, and skewness of the *N* permutation replicates obtained above by *ζ*_1_, *ζ*_2_, and *ζ*_3_, respectively. Then by matching them with the three corresponding theoretical moments, we have the following three equations as


$$F(z)=\text{exp}\;\{-1+{\left(\frac{z-\eta }{\alpha }\right)}^{\frac{1}{\kappa }}\kappa \},z\in R,$$

$$\{\begin{array}{c} \zeta_{1}=\eta +\frac{\alpha }{\kappa }[1-\Gamma (1+\kappa )],\\ \zeta_{2}={\left(\frac{\alpha }{\kappa }\right)}^{2}[\Gamma (1+2\kappa )-\Gamma^{2}(1+\kappa )],\\ \zeta_{3}=\frac{\kappa }{\left|\kappa \right|}\{\frac{-\Gamma (1+3\kappa )+3\Gamma (1+\kappa )\Gamma (1+2\kappa )-2\Gamma^{3}(1+\kappa )}{{\left[\Gamma (1+2\kappa )-\Gamma^{2}(1+\kappa )\right]}^{3/2}}\}. \end{array}$$

Then parameters κ,α, and *η* can be estimated by solving these equations and can be denoted as $\hat{\kappa },\;\hat{\alpha },$ and $\hat{\eta }$, respectively. Given the observed value of the MaxKAT, *t*, its *P*-value is calculated by plugging the estimates $\hat{\kappa },\;\hat{\alpha },$ and $\hat{\eta }$ into 1−F(t).

Note that there exist some inference methods for KAT-type statistics that are free from doing permutations, such as the KRV method proposed by Zhan *et al.* (2017a). These methods can be used to approximate the distribution of KAT and wKAT. However, they are not applicable to the distributional approximation of MaxKAT, since MaxKAT is a maximum-type statistic, whose moments are very complicated to calculate. So we use the permutation strategy in this work.

As described above, the significance of MaxKAT can be calculated through two procedures, the permutation strategy and the GEV distribution approximation. We denote them as MaxKAT.perm and MaxKAT.gev, respectively. Though both MaxKAT.perm and MaxKAT.gev rely on the permutation strategy to calculate *P*-values, MaxKAT.gev has a computational advantage over MaxKAT.perm. That is, MaxKAT.gev allows to obtain *P*-values that are arbitrarily small, which will reduce computational cost greatly when the significance level is stringent. For example, when the number of permutation is 1000, the minimum *P*-value that MaxKAT.perm can get is 0.001, while the minimum *P*-value that MaxKAT.gev can get is arbitrarily small. Under this condition, when the significance level is stringent, such as $1\times 10^{-6}$, the number of permutation for MaxKAT.perm needs to be no less than $1\times 10^{6}$ to obtain meaningful results, but that for MaxKAT.gev can still be 1000. We conduct simulation to demonstrate this point in Section 3.2.

### 2.3 Choices of kernel functions

Here, we list some commonly used kernel functions in pleiotropic genetic association study. The inner product-based kernel (Price *et al.* 2006) and the identity-by-state (IBS) kernel for genotype data are

1. Inner product-based kernel: $k(x_{1},x_{2})=\frac{1}{m}\sum_{r=1}^{m}{\bar{x}}_{1r}{\bar{x}}_{2r}$, where ${\bar{x}}_{1r}=(x_{1r}-2p_{r})/\sqrt{p_{r}(1-p_{r})}$ and the allele frequency *p_r_* can be estimated via ${\hat{p}}_{r}=(1/2n)\sum_{i=1}^{n}x_{ir}$,
2. IBS kernel: $k(x_{1},x_{2})=(1/2m)\sum_{r=1}^{m}\left(2-|x_{1r}-x_{2r}|\right)$.

For phenotypes, the commonly used kernel functions are

1. Gaussian kernel: $\tilde{k}(y_{1},y_{2};h)=\text{exp}\;\{-\sum_{r=1}^{\tilde{m}}{\left(y_{1r}-y_{2r}\right)}^{2}/h\}$,
2. Polynomial kernel: $\tilde{k}(y_{1},y_{2};h)=(1/2\tilde{m})\sum_{r=1}^{\tilde{m}}{\left(y_{1r}y_{2r}+1\right)}^{h}$, and
3. Binary kernel: $\tilde{k}(y_{1},y_{2})=\sum_{r=1}^{\tilde{m}}I(y_{1r}=y_{2r})$.

When phenotypes are of mixture type, i.e. including both continuous and binary traits, we can use two types of kernel to form a mixture one.

When there are covariates, such as sex, age, and population stratification confounders, to be adjusted for, we can first regress phenotypes on covariates to obtain residuals, which can be treated as “new phenotypes”, and then use these “new phenotypes” in the subsequent association analysis. Applications of this strategy are illustrated in the later data analysis.

## 3 Simulation studies

In this section, we conduct simulations to evaluate MaxKAT from the perspective of the type I error rates and powers, computational time and kernel function choice.

### 3.1 Type I error rates and powers

We conduct extensive simulations to demonstrate the type I error rates and powers of MaxKAT by comparing with KAT. The sample size is set to 100. And the dimensions of genotypes and phenotypes are, respectively, chosen from {20,50,100,200,500}. To generate genotypes and phenotypes, we first sample latent variables $z_{i1}$ and $z_{i2}$ from the multivariate normal distribution $N(1_{50},I_{50})$, and then produce $x_{i}$ and $y_{i}$ based on $z_{i1}$ and $z_{i2}$, respectively. To be specific, $x_{i}$ is sampled from $N(z_{i1}^{⊤}\beta_{x},\Delta_{x})$ and $y_{i}$ is generated from the following model where function f(·) is set to be element-wise for simplicity. We set three types of models to generate $y_{i}$:


$$y_{i}=f{\left(z_{i2}\right)}^{⊤}\beta_{y}+\epsilon_{i},$$

1. Model I: $f(z_{i})=z_{i},\;\epsilon_{i}∼N(0,\Delta_{y})$,
2. Model II: $f(z_{i})=\text{exp}(z_{i}),\;\epsilon_{i}∼N(0,\Delta_{y}),$ and
3. Model III: $f(z_{i})=z_{i},\;\;\text{log}(\epsilon_{i})∼N(0,\Delta_{y})$.

Since the association strengths between pairs of genotype decrease as their physical distances increase, Δ_*x*_ is set to have autoregressive structure with its (*i*, *j*)th element being $\rho^{\left|i-j\right|}$. To model different association strengths within multiple phenotypes, two covariance structures are considered for Δ_*y*_: the autoregressive structure mentioned above and the compound-symmetry structure $\Delta_{y}=(1-\rho )I_{\tilde{m}}+\rho 1_{\tilde{m}}1_{\tilde{m}}^{⊤}$. We set *ρ* to be 0.1, 0.5, and 0.9 to indicate diverse association strengths. For the sake of simplicity, parameter matrices *β_x_* and *β_y_* are set to be equal constant matrices, and the values of their elements *β* are listed in Table 1. To mimic the coding of genotype data which is coded as 0, 1, and 2, $x_{i}$ is transferred to 0, 1, and 2 based on two quantile values of ${\left(1-q\right)}^{2}$ and $1-q^{2}$ to consider the Hardy–Weinberg equilibrium, where *q *=* *0.3 is the minor allele frequency. The recoded $x_{i}$ is still denoted as $x_{i}$ for the notational simplicity. Under the null hypothesis of no association, $z_{i1}$ and $z_{i2}$ are sampled independently so that *x_i_* and *y_i_* are independent. When the alternative hypothesis is true, $z_{i1}$ and $z_{i2}$ are set to be equal.

**Table 1.** The value of *β* for different scenarios.

Δ_*y*_	Autoregressive			Compound symmetry		
*Ρ*	0.1	0.5	0.9	0.1	0.5	0.9
Model I	0.6	0.8	1	1	1.8	2.5
Model II	0.6	0.6	1	0.8	1.3	2
Model III	1.1	1.3	1.8	1.5	1.9	2.5

We apply both MaxKAT.perm and MaxKAT.gev to the simulation study to investigate the accuracy of GEV approximation strategy. The significance of KAT is also calculated via permutation procedure to make the results comparable. For each scenario, 1000 repetitions are conducted and the type I error rates and powers are obtained by calculating the proportions of *P*-values less than the significance level of 0.05. Given each dataset, we use the inner product-based kernel (Price et al. 2006) described above to calculate genetic similarities and use the Gaussian kernel to calculate phenotypic similarities.

**Figure 1.**
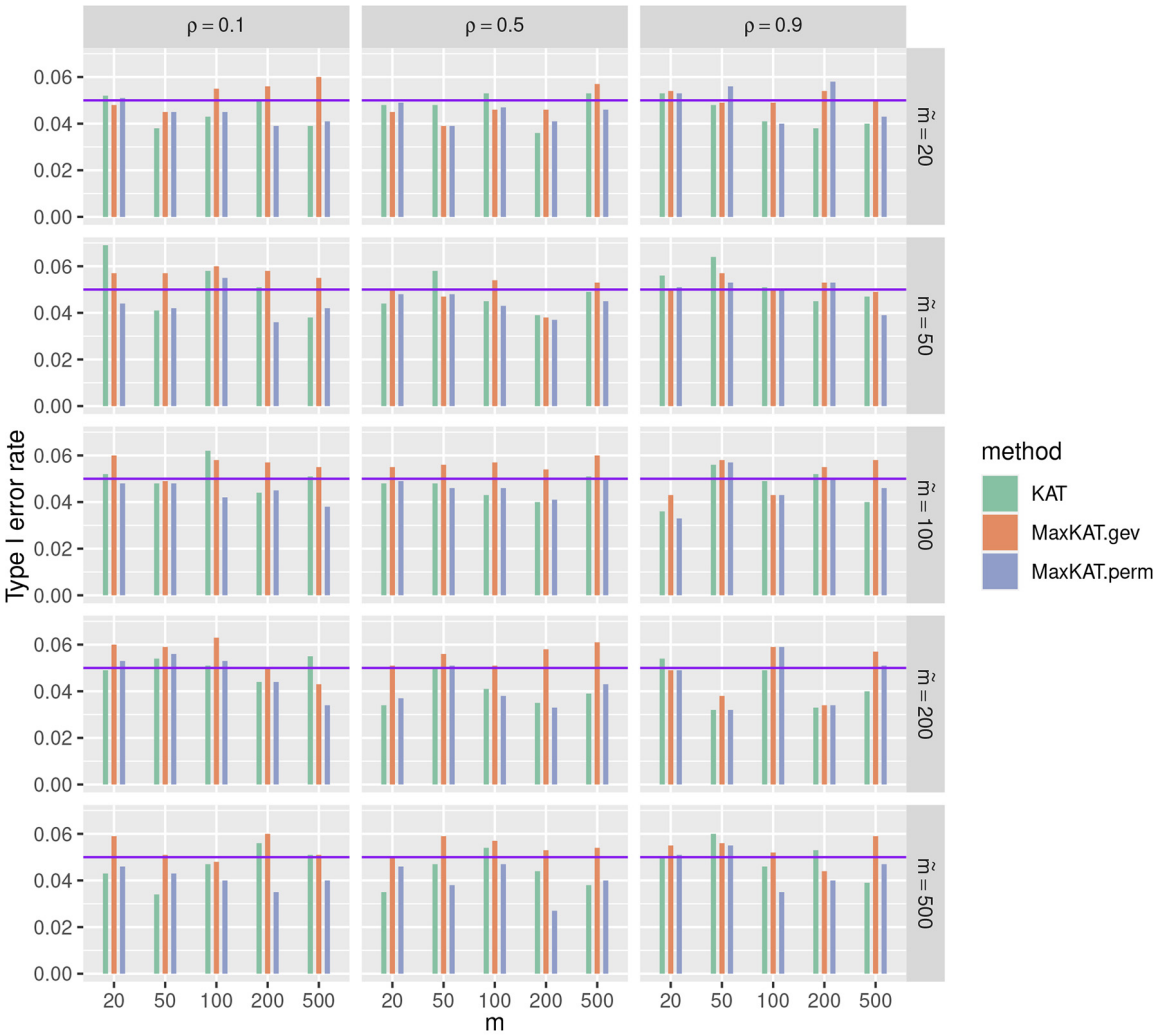
Barplots for the type I error rates of KAT, MaxKAT.perm, and MaxKAT.gev under Model I when Δ_*y*_ is of autoregressive structure.

**Figure 2.**
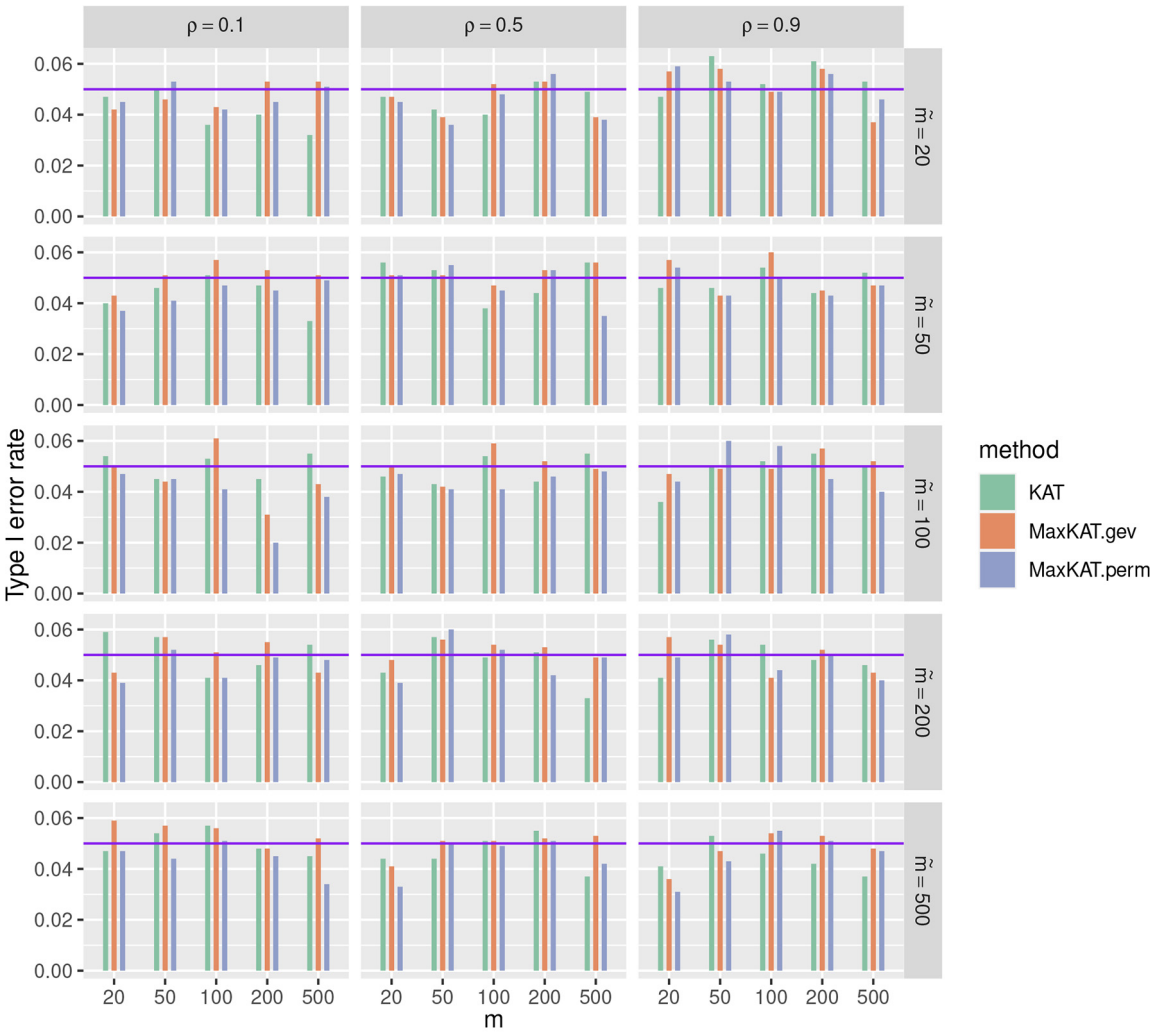
Barplots for the type I error rates of KAT, MaxKAT.perm, and MaxKAT.gev under Model I when Δ_*y*_ is of compound-symmetry structure.

The type I error rates are presented in Figs 1 and 2, and Supplementary Figs S1–S4 in the Supplementary Materials. The purple horizontal line corresponding to 0.05 in each plot is drawn for better reference. It can be seen that all these three methods KAT, MaxKAT.perm, and MaxKAT.gev can control the type I error rates properly, with their *P*-values fluctuating around the level of 0.05. For example, when Δ_*y*_ is of autoregressive structure (Fig. 1), ρ=0.9 and $\tilde{m}=100$, the *P*-values of KAT are 0.036, 0.056, 0.049, 0.052, and 0.040 as *m* increases from 20 to 500, and the corresponding *P*-values of MaxKAT.perm (MaxKAT.gev) are 0.033 (0.043), 0.057 (0.058), 0.043 (0.043), 0.050 (0.055), and 0.046 (0.058), respectively.

**Figure 3.**
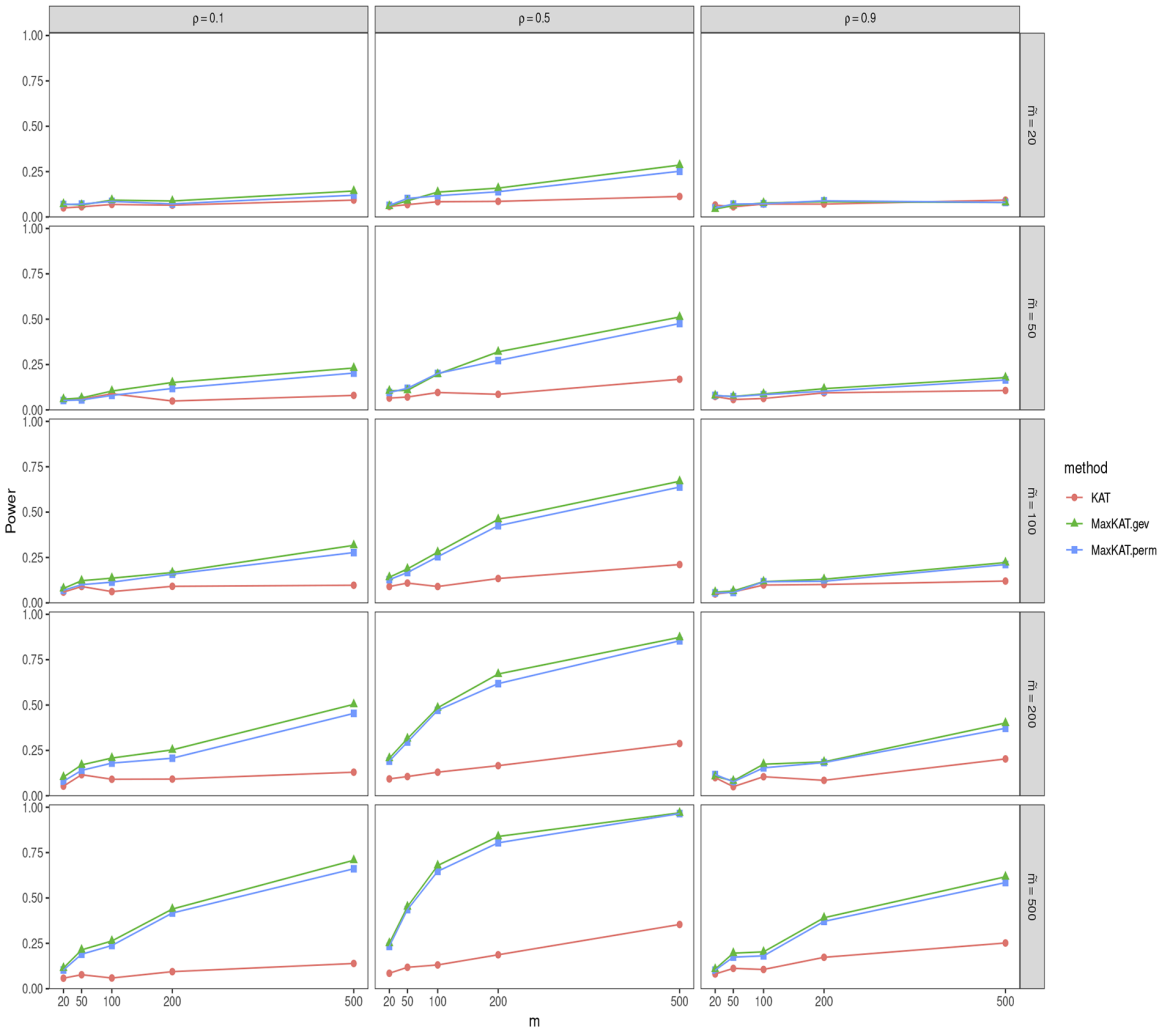
The empirical powers of KAT, MaxKAT.perm, and MaxKAT.gev under Model I when Δ_*y*_ is of autoregressive structure.

**Figure 4.**
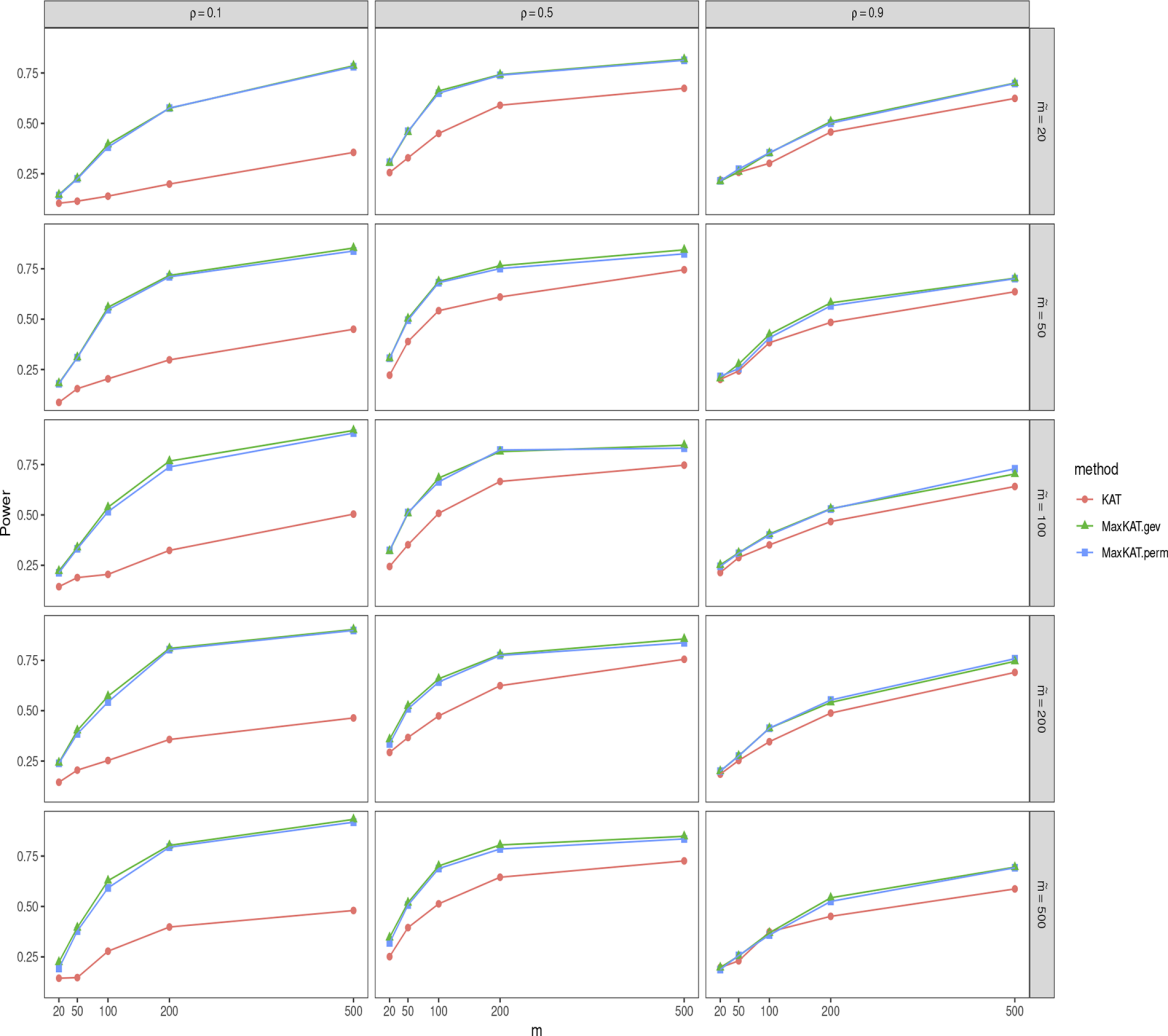
The empirical powers of KAT, MaxKAT.perm, and MaxKAT.gev under Model I when Δ_*y*_ is of compound-symmetry structure.

The empirical powers are shown in Figs 3 and 4, and Supplementary Figs S5–S8 in the Supplementary Materials. Take Fig. 4 as an example. In this figure, the powers of MaxKAT.perm and MaxKAT.gev are close, and they both are higher than that of KAT in all the considered scenarios. MaxKAT has higher powers even when the data are of high dimension. To be specific, as the dimension *m* increases, the power gain of MaxKAT relative to KAT increases as well, indicating that the advantage of MaxKAT over KAT is enlarged in high-dimension settings. For example, in Fig. 4, given ρ=0.1 and $\tilde{m}=500$, the power gain of MaxKAT.perm over KAT increases as *m* increases. When *m *=* *20, the empirical power of KAT is 0.144, while that of MaxKAT.perm is 0.192. When *m *=* *100, the respective powers of KAT and MaxKAT.perm are 0.278 and 0.579. When *m* is as large as 500, the powers of KAT and MaxKAT.perm are 0.480 and 0.918, respectively, with the latter having a power gain as large as 0.438. The results in Supplementary Figs S5 and S6 further demonstrate that MaxKAT excels over KAT in diverse relationships (nonlinear relationships for example), and those in Supplementary Figs S7 and S8 demonstrate the advantages of MaxKAT in diverse distributions (lognormal distribution for example).

**Figure 5.**
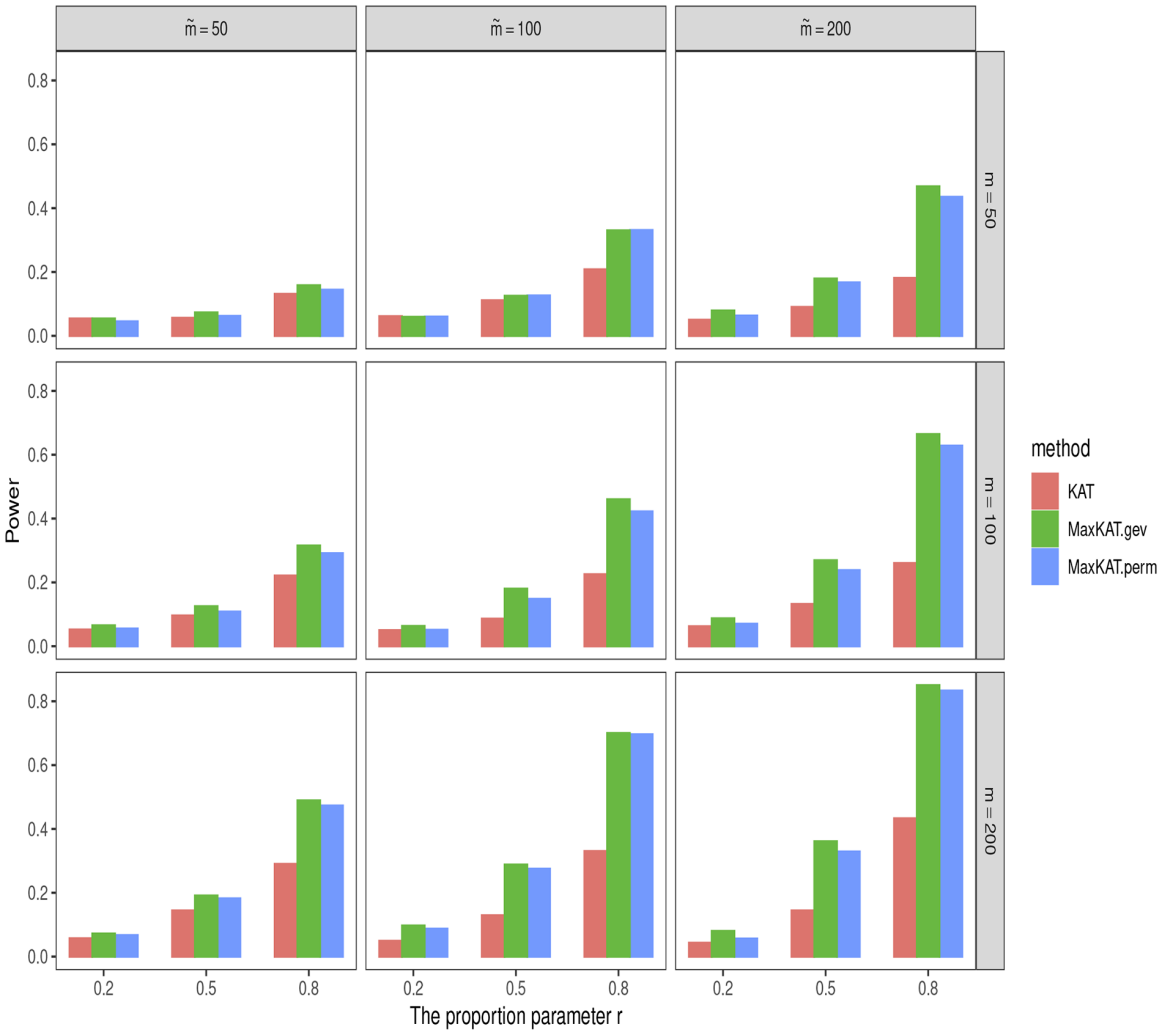
Barplots for powers of KAT, MaxKAT.perm, and MaxKAT.gev when variables are partially related under autoregressive structure of Δ_*y*_.

**Figure 6.**
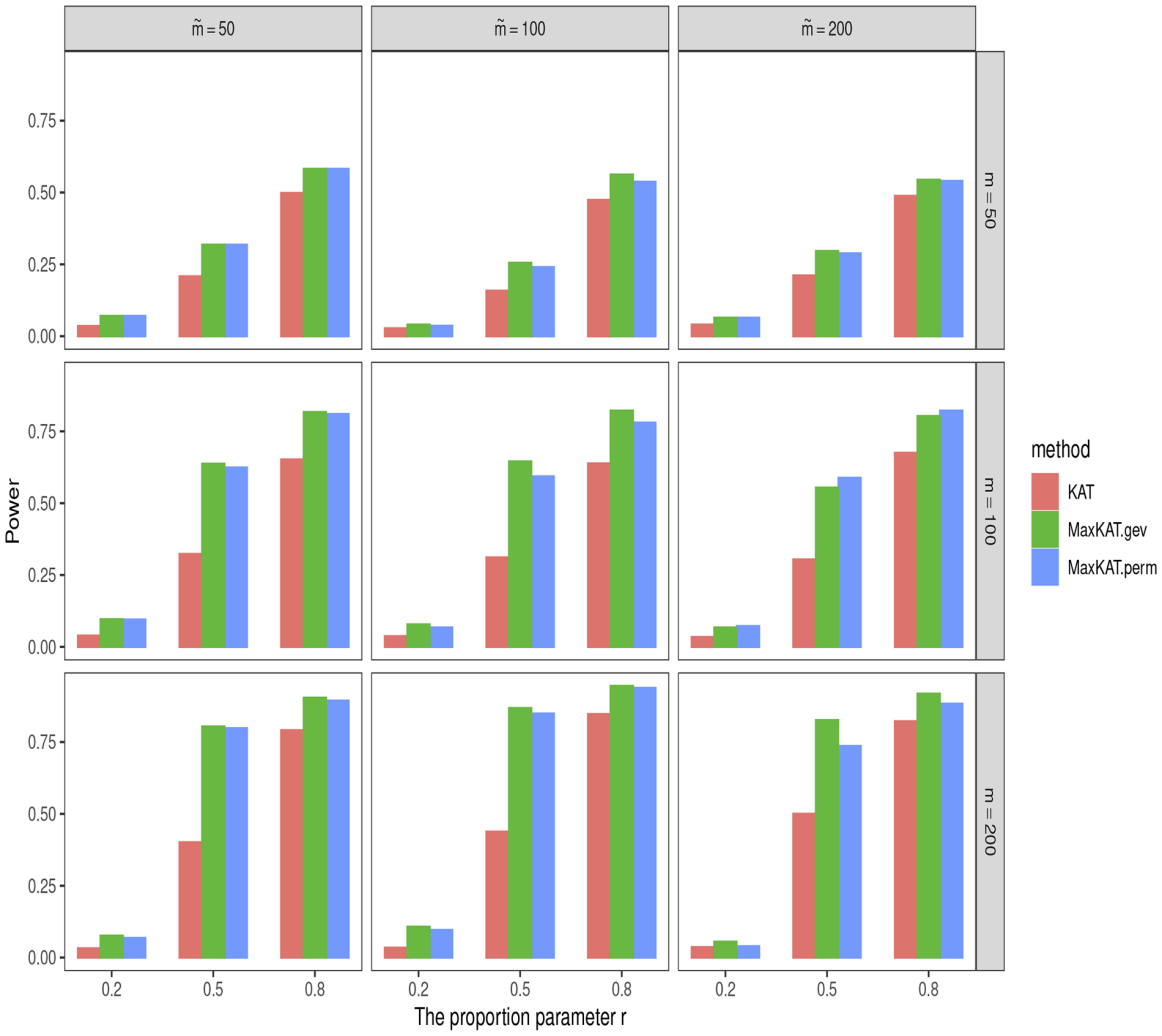
Barplots for powers of KAT, MaxKAT.perm, and MaxKAT.gev when variables are partially related under compound-symmetry structure of Δ_*y*_.

We also consider situations with different association patterns. The dimensions *m* and $\tilde{m}$ are chosen from {50, 100, 200}. Phenotypes are generated via Model I. On one hand, to consider situations where multiple genetic variables and multiple phenotypes are partially related, we respectively let a proportion of *r* elements in $x_{i}$ and $y_{i}$ be related to the potential variants, with proportion *r* being chosen from {20%,50%,80%}. On the other hand, to consider different effect sizes, non-zero values in *β_x_* and *β_y_* are generated from the uniform distribution Unif(0.5,1.5) for autoregressive $\Delta_{y}(\rho =0.5)$ and Unif(1.5,2.5) for compound symmetry $\Delta_{y}(\rho =0.5)$. The bar plots for powers of different methods are shown in Figs 5 and 6. These results show that when the variables are partially related to different effect sizes, the newly proposed MaxKAT still outperforms KAT in detecting association relationships.

### 3.2 Computational time comparison

As described in Section 2.2, the GEV-based MaxKAT.gev has a computational advantage over MaxKAT.perm and KAT when the significance level is stringent. We conduct simulations to illustrate this point. Since MaxKAT.perm and KAT calculate *P*-values through the permutation strategy, the number of permutation *N* increases as the significance level gets more stringent. For example, *N *=* *1000 is sufficient when the significance level is $5\times 10^{-2}$, but *N* needs to be no less than $1\times 10^{6}$ when the significance level is set to be $1\times 10^{-6}$. On the contrary, 1000 is always sufficient to calculate *P*-values for MaxKAT.gev, no matter how stringent the significance level is. We let *N* choose values from $\left\{1\times 10^{3},1\times 10^{4},1\times 10^{5},1\times 10^{6}\right\}$ for KAT and MaxKAT.perm, and keep *N* for MaxKAT.gev to be 1000, to investigate their run time. Dimensions *m* and $\tilde{m}$ are set to be equal, taking values from {50, 100, 200}. And phenotypes are generated via Model I with compound symmetry $\Delta_{y}(\rho =0.5)$. In each scenario, the repetition time is 10 to calculate the average running time for each method. The results are presented in Table 2.

**Table 2.** The average running time (measured by seconds) of each method under different scenarios.

Method	KAT				MaxKAT.perm				MaxKAT.gev
*N*	$1\times 10^{3}$	$1\times 10^{4}$	$1\times 10^{5}$	$1\times 10^{6}$	$1\times 10^{3}$	$1\times 10^{4}$	$1\times 10^{5}$	$1\times 10^{6}$	$1\times 10^{3}$
$m=\tilde{m}=50$	0.08	0.78	7.57	83.36	20.74	206.73	2296.8	20 505.97	20.73
$m=\tilde{m}=100$	0.1	0.7	6.99	78.46	36.86	354.08	3447.84	34 854.47	36.94
$m=\tilde{m}=200$	0.08	0.71	6.87	69.7	37.28	359.76	3410.68	34 688.64	37.24

Just as expected, the computational time for KAT and MaxKAT.perm increases as *N* gets longer, i.e. as the significance level gets more stringent. And MaxKAT.perm is much more time-consuming than MaxKAT.gev as *N* increases, demonstrating the advantage of GEV approximation in situations with stringent significance levels. Though KAT is more time-saving than MaxKAT.gev when *N* is no larger than $1\times 10^{5}$, the running time of MaxKAT.gev is acceptable and it achieves much higher powers, which has been demonstrated before. And the running time of KAT excels that of MaxKAT.gev when *N* is $1\times 10^{6}$, verifying the advantage of MaxKAT.gev further under conditions of stringent significance level.

### 3.3 Comparison of different choices on kernel functions

Note that although we use the inner product-based kernel (for genetic data) and Gaussian kernel (for phenotypic data) in the above analysis, other types of kernels can also be used. We conduct simulations to investigate the effect of kernel choice on the performances of different methods. Four types of kernel choice are considered:

- Apply inner product-based kernel to genetic data, and Gaussian kernel to phenotypic data.
- Apply IBS kernel to genetic data, and Gaussian kernel to phenotypic data.
- Apply inner product-based kernel to genetic data, and polynomial kernel to phenotypic data.
- Apply IBS kernel to genetic data, and polynomial kernel to phenotypic data.

**Figure 7.**
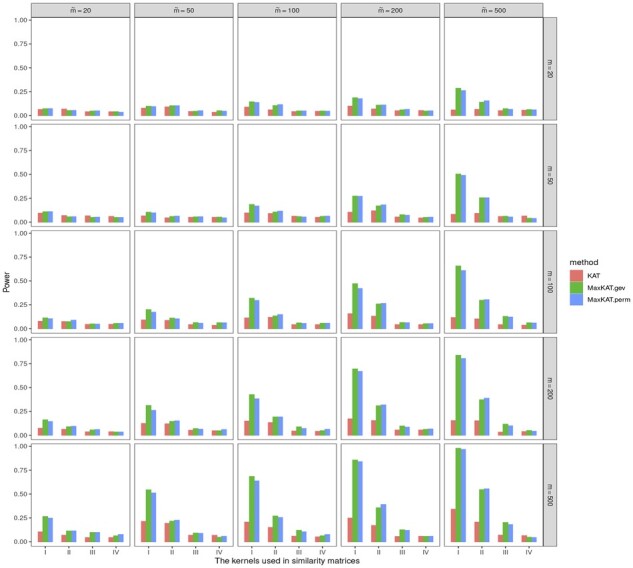
Barplots for powers of KAT, MaxKAT.perm, and MaxKAT.gev with different kernel choices under autoregressive structure of Δ_*y*_.

**Figure 8.**
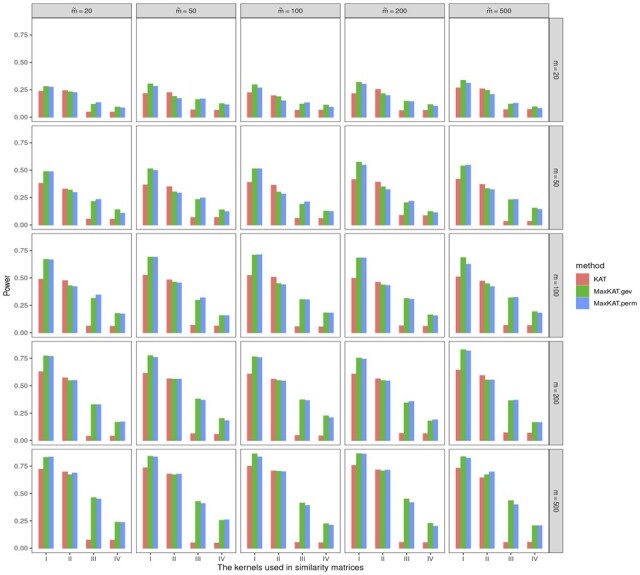
Barplots for powers of KAT, MaxKAT.perm, and MaxKAT.gev with different kernel choices under compound-symmetry structure of Δ_*y*_.

Data are generated via Model I. And the powers of different methods for autoregressive $\Delta_{y}(\rho =0.5)$ and compound symmetry $\Delta_{y}(\rho =0.5)$ are displayed in Figs 7 and 8, respectively. It can be seen that the choices of kernel functions do have a remarkable effect on powers of all the methods, and the combination of inner product-based kernel and Gaussian kernel performs better than other kernels in the scenarios considered here. Since how to choose kernel functions is not the focus of our attention, we omit this issue in the present work and leave it to further study. However, it is noteworthy that although the powers vary with different kernel functions, MaxKAT outperforms KAT in all conditions with diverse kernel functions.

## 4 Applications to biomedical porcine datasets

Serum lipids are important indicators for health status of humans. Their abnormal concentrations are associated with cardiovascular disease and diabetes. In clinical tests, six blood lipid traits, including total cholesterol (TC), triglycerides (TG), atherosclerosis index (AI), low-density lipoprotein cholesterol (LDL-C), high-density lipoprotein (HDL-C), and the ratio of the HDL-C and LDL-C (denoted as HDL-C/LDL-C), are widely used in risk assessment of cardiovascular disease. Locating genetic variants regulating these traits might be useful for the prevention, identification, and treatment of the disease.

Pigs have been used as biomedical experimental materials of human disease research for decades. Yang et al. (2015) genotyped 61 565 SNPs of the pig genome and conducted GWAS on these six blood lipid traits to identify the trait-related SNPs. In this work, we apply KAT and MaxKAT to the Erhualian pig dataset in Yang et al. (2015) to compare their performances. Erhualian is a Chinese indigenous pig breed which is well known for its high prolificacy. We first preprocess the data using quality control. Three hundred and thirty-three pigs are genotyped on 61 565 SNPs and phenotyped on the 6 blood lipid traits mentioned above. After removing observations with missing trait values, data from 322 pigs remain. Quality control is also conducted on genotypes. Specifically, only SNPs with missing value proportion being <5%, minor allele frequency being larger than 1%, and minimum genotype cell number being larger than 5 can be used for further analysis. So 20 687 SNPs remain to be tested.

We apply multidimensional scaling (MDS) (Li and Yu 2008) to the covariance information of the SNP data to correct for population stratification, which is implemented in the R package *AssocTests* (Wang et al. 2020). Ten principal coordinates are generated and treated as confounder covariates. Six traits are regressed on these covariates to remove their effects, and the residuals are used to calculate the similarity matrix via the Gaussian kernel function. The inner product-based kernel (Price et al. 2006) is used to calculate the genetic similarity matrix. *θ* and $\tilde{\theta }$ are set to take on values in $\left\{\frac{1}{2},\frac{3}{4},1,2,3\right\}$ to calculate MaxKAT. The *P*-values of MaxKAT.perm and MaxKAT.gev are 0.008 and 0.009, respectively, while KAT gives a *P*-value of 0.072. These values indicate that MaxKAT detects the association relationship between phenotypes and genotypes, whereas KAT misses it.

MaxKAT and KAT can also be used in association analysis based on SNP regions, such as genes, pathways, and SNP intervals. In the GWAS analysis of Yang et al. (2015), eight SNPs of Erhualian are identified to be related to the phenotypes of interest. To compare the performances of MaxKAT and KAT in the SNP region-based analysis, we apply both methods to SNP intervals of length 3 Mb that are, respectively, centered at these SNPs. First, we conduct the same quality control procedure as described before in these intervals. The numbers of SNPs in these SNP intervals before and after quality control (denoted as *m*_1_ and *m*_2_, respectively) are presented in Table 3. For each SNP interval, we use SNPs in the corresponding interval to calculate the genotypic similarity matrix via the inner product-based kernel function. The phenotypic similarity matrix is the same as that used before. The resulting *P*-values for all eight SNP intervals are shown in Table 3. It can be seen that both KAT and MaxKAT detect the association relationships of the first two SNP intervals, but they do not identify the association relationships of the rest SNP intervals except for the fifth SNP interval. This is because that these SNPs are identified by Yang et al. (2015) via GWAS analysis based on single trait and single SNP, while KAT and MaxKAT conduct association analysis based on multiple traits and multiple SNPs, where association signals may be weakened when multiple traits and multiple SNPs are partially correlated. However, as for the fifth SNP interval, the *P*-values of KAT MaxKAT.perm and MaxKAT.gev are 0.020 and 0.023, respectively, confirming the finding reported in Yang *et al.* (2015). In contrast, the *P*-value obtained using KAT is 0.065, indicating a failure to detect this association relationship. These results suggest that MaxKAT performs better than KAT in identifying association signals based on SNP regions.

**Table 3.** Results on *P*-values of association analysis based on SNP intervals.

Chromosome	Position	Illumina SNP	*m*_1_	*m*_2_	KAT	MaxKAT.perm	MaxKAT.gev
1	94111766	MARC0031521	62	20	0.010	0.006	0.004
1	95099817	ASGA0003711	58	19	0.011	0.009	0.008
5	34643398	ASGA0025331	77	33	0.485	0.201	0.188
7	7473615	ASGA0030933	82	28	0.073	0.062	0.059
9	14306925	MARC0046646	76	37	0.065	0.020	0.023
18	59827202	ASGA0085659	41	4	0.583	0.679	0.681
18	59833467	ASGA0100953	41	4	0.614	0.648	0.645
18	59971337	MARC0037227	37	4	0.589	0.638	0.659

## 5 Discussion

Association testing methods being free of constraints on data dimension and data structure are in great demand nowadays in the big data era. The kernel-based approach is a good alternative to this issue and has been generally recommended (Chen *et al.* 2016, Lee *et al.* 2017, Zhan *et al.* 2017a,b). However, it suffers from substantial power loss when the correlation among phenotypes is moderate to strong. To handle this problem, we propose a maximum type kernel-based association test, MaxKAT, and use a generalized extreme value distribution to appropriate its distribution. Simulation studies and real data analysis demonstrate the superiority of MaxKAT over KAT, verifying its efficiency and sensitivity in detecting pleiotropic genetic effects for multiple phenotypes.

As MaxKAT relies on a permutation procedure to calculate its *P*-value, it takes more time to implement MaxKAT in larger sample sizes. However, compared with distributional approximation-based methods, this permutation-based method is free from requirement on the sample size and has high powers even when sample sizes are small. Besides, given that MaxKAT boosts the power in detecting association relationships remarkably, its time cost is acceptable.

Although MaxKAT is developed for genetic pleiotropy study, it can also be applied to other areas, such as microbiome data analysis and genomic study, where association analysis between microbiome composition and phenotypes, and between gene expression levels and genetic variants are conducted, respectively. We would like to point out that MaxKAT can also be used to handle data with more complex structures such as tree, network, and image, only if a reasonable kernel is available. The R codes for the implementation of MaxKAT are assembled in the R package named *MaxKAT* which is posted on GitHub https://github.com/WangJJ-xrk/MaxKAT.

## Supplementary Material

btad291_Supplementary_Data

## Data Availability

The data used in case study are available at https://datadryad.org/stash/dataset/doi:10.5061\%2Fdryad.4gh70.
