# Supplementary material for: A maximum kernel-based association test to detect the pleiotropic genetic effects on multiple phenotypes
Source: Bioinformatics. 2023 Apr 27;39(5):btad291. doi: 10.1093/bioinformatics/btad291 (PMC10174706; doi:10.1093/bioinformatics/btad291)
Supplement: btad291_Supplementary_Data [file btad291_supplementary_data.pdf]

Supplemental Materials for "A maximum kernel-based association test to detect the pleiotropic genetic effects on multiple phenotypes"

This Supplemental Materials contain three sections. Section 1 demonstrates the perfomance of wKAT$(\theta, \tilde{\theta})$ to shed some light on the motivation of wKAT$(\theta, \tilde{\theta})$ and MaxKAT. And the asymptotic properties of wKAT$(\theta, \tilde{\theta})$ and MaxKAT and their proofs are presented in Section 2. The supplemental figures are displayed in Section 3. In Section 4, the R codes for the implementation of MaxKAT are documented.

1 Simulation studies for wKAT$(\theta, \tilde{\theta})$

To demonstrate the performances of wKAT$(\theta, \tilde{\theta})$ on different choices of θ and $\tilde{\theta}$, and show the motivation of our method, we choose θ and $\tilde{\theta}$ from $\{\frac{1}{2}, \frac{3}{4}, 1, 2, 3\}$ and conduct simulation studies to compare their empirical powers. The sample size n is set to be 100. And the dimensions m and $\tilde{m}$ are set to be 50 and 100, respectively. Genotypes $\boldsymbol{x}_i$s are sampled from $N(\mathbf{0}_m, 0.9\mathbf{I}_m)$ and the corresponding phenptype $\boldsymbol{y}_i$ is generated from $N(\boldsymbol{x}_i^\top \beta, 0.1\mathbf{I}_{\tilde{m}} + 0.9\mathbf{1}_{\tilde{m}}\mathbf{1}_{\tilde{m}}^\top)$, where β is a constant matrix with all its elements being 0.8. And powers of wKAT$(\theta, \tilde{\theta})$ for different θs and $\tilde{\theta}$s are presented in Table S1. Given $\theta = 1$, the powers of wKAT$(1, 1/2)$, wKAT$(1, 3/4)$, wKAT$(1, 1)$, wKAT$(1, 2)$, and wKAT$(1, 3)$ are 0.873, 0.886, 0.883, 0.877, and 0.871, respectively, with the most powerful one being obtained when $\tilde{\theta} < 1$. While given $\tilde{\theta} = 1$, the powers of wKAT$(1/2, 1)$, wKAT$(3/4, 1)$, wKAT$(1, 1)$, wKAT$(2, 1)$, and wKAT$(3, 1)$ are 0.822, 0.861, 0.883, 0.903, and 0.909, respectively, demonstrating an increasing trend as θ increases. Among all the wKAT$(\theta, \tilde{\theta})$s, the most powerful one is wKAT$(3, 1/2)$. All these results indicate that when the association relationships are weak within multiple variates (such as $\boldsymbol{x}_i$ in this simulation), index (such as θ) larger than 1 helps improve the power of wKAT. While when the association relationships are strong within variables (such as $\boldsymbol{y}_i$ in this simulation), taking index value (such as $\tilde{\theta}$) less than 1 may boost the power.

2 Asymptotic properties of wKAT$(\theta, \tilde{\theta})$s and MaxKAT

We develop the theoretical properties of wKAT$(\theta, \tilde{\theta})$ and MaxKAT. Without loss of generality, we assume that $\Theta = \tilde{\Theta} = [\epsilon_0, \infty)$, with ϵ_0 being a small positive real number. Denote the density function of $\mathbf{X}$ by $p(\boldsymbol{x})$, and the centered version of the kernel function $k(\boldsymbol{x}_1, \boldsymbol{x}_2)$ by $k_c(\boldsymbol{x}_1, \boldsymbol{x}_2) \triangleq k(\boldsymbol{x}_1, \boldsymbol{x}_2) - E_{\boldsymbol{x}_1}(k(\boldsymbol{x}_1, \boldsymbol{x}_2)) - E_{\boldsymbol{x}_2}(k(\boldsymbol{x}_1, \boldsymbol{x}_2)) + E(k(\boldsymbol{x}_1, \boldsymbol{x}_2))$. Let $\lambda_i^*, i = 1, 2, \cdots$

Table S1: The powers of wKAT$(\theta, \tilde{\theta})$

θ \ $\tilde{\theta}$	$\frac{1}{2}$	$\frac{3}{4}$	1	2	3
$\frac{1}{2}$	0.816	0.833	0.822	0.826	0.828
$\frac{3}{4}$	0.857	0.858	0.861	0.857	0.855
1	0.873	0.886	0.883	0.877	0.871
2	0.904	0.907	0.903	0.903	0.903
3	0.912	0.909	0.909	0.902	0.903

be the solutions to equations $\int k_c(\boldsymbol{x}_1, \boldsymbol{x}_2) u_i(\boldsymbol{x}_1) p(\boldsymbol{x}_1) d\boldsymbol{x}_1 = \lambda_i^* u_i(\boldsymbol{x}_2), i = 1, 2, \cdots$ in a non-ascending order, that is, λ_i^*s are the eigenvalues of the kernel function $k_c(\boldsymbol{x}_1, \boldsymbol{x}_2)$ and $u_i(\boldsymbol{x})$s are the corresponding eigenfunctions. Similarly, we can denote eigenvalues $\tilde{\lambda}_j^*, j = 1, 2, \cdots$ and eigenfunctions $\tilde{u}_j(\boldsymbol{y}), j = 1, 2, \cdots$ for the centered version of the kernel function $\tilde{k}(\boldsymbol{y}_1, \boldsymbol{y}_2)$.

To derive the asymptotic distribution of wKAT$(\theta, \tilde{\theta})$, we first list two assumptions and two lemmas. Throughout the paper, let the symbol "$\xrightarrow{d}$" represent "converges in distribution" and "$\xrightarrow{p}$" represent "converges in probability".

Assumption 1 *Assume the kernel functions $k(\boldsymbol{x}_1, \boldsymbol{x}_2)$ and $\tilde{k}(\boldsymbol{y}_1, \boldsymbol{y}_2)$ are symmetric and continuous.*

Assumption 2 *There exist $0 < \theta_0, \tilde{\theta}_0 < 1$ satisfying $\sum_{i=1}^{\infty} \left|(\lambda_i/n)^{\theta_0} - (\lambda_i^*)^{\theta_0}\right| \xrightarrow{p} 0$ and $\sum_{j=1}^{\infty} \left|(\tilde{\lambda}_j/n)^{\tilde{\theta}_0} - (\tilde{\lambda}_j^*)^{\tilde{\theta}_0}\right| \xrightarrow{p} 0$, as $n \to \infty$.*

Remark 1 *Assumption 1 is easily satisfied since the commonly used kernel functions such as linear kernels and Gaussian kernels are always symmetric and continuous. Assumption 2 ensures a convergence condition for the test statistic wKAT$(\theta, \tilde{\theta})$. Zhang* et al. *(2012) showed that $\lambda_i/n \xrightarrow{p} \lambda_i^*$, $i = 1, 2, \cdots, n$. Then, $(\lambda_i/n)^\theta \xrightarrow{p} (\lambda_i^*)^\theta$, for any $\theta > 0$. Assumption 2 requires that the sum of absolute differences $|(\lambda_i/n)^\theta - (\lambda_i^*)^\theta|$ approaches zero in probability. Similar assumptions also hold for $\tilde{\lambda}_j, \tilde{\lambda}_j^*, j = 1, \cdots, n$.*

Lemma 1 *If the kernel function $k(\boldsymbol{x}_1, \boldsymbol{x}_2)$ is symmetric, and its vth derivative $\frac{\partial k^v(\boldsymbol{x}_1, \boldsymbol{x}_2)}{\partial^v \boldsymbol{x}_1}$ exists and is continuous, then for any constant θ satisfying $\theta(v + \frac{1}{2}) > 1$, we have,*

$$\sum_{i=1}^{\infty} (\lambda_i^*)^\theta < \infty.$$

Proof. Following the results of Weyl (1912), the eigenvalues of $k(\boldsymbol{x}_1, \boldsymbol{x}_2)$ decays at the order $|\lambda_i^*| = o(i^{-v-1/2})$. Thus if $\theta(v + \frac{1}{2}) > 1$, the p series $\sum_i^\infty i^{-\theta(v+\frac{1}{2})}$ converges. Then we have $\sum_{i=1}^\infty (\lambda_i^*)^\theta < \infty$.

Remark 2 *Lemma 1 shows the finiteness for summation of eigenvalues at the θth power given the continuity of the vth partial derivative for the kernel function. Note that the Gaussian kernel $k(x_i, x_j; h) = \exp\{-\sum_{l=1}^m (x_{il} - x_{jl})^2/h\}$ satisfies the continuity condition at any order of its derivative. So if it is used in calculating $\mathbf{S}$ and $\tilde{\mathbf{S}}$, we have $\sum_{i=1}^\infty (\lambda_i^*)^\theta < \infty$ and $\sum_{j=1}^\infty (\tilde{\lambda}_j^*)^{\tilde{\theta}} < \infty$ for any positive real numbers θ and $\tilde{\theta}$. Given the kernels used, we choose Θ and $\tilde{\Theta}$ such that Lemma 1 is satisfied to ensure the asymptotic properties of wKAT$(\theta, \tilde{\theta})$. Similar assumption with $\sum_{i=1}^\infty (\lambda_i^*)^{1/2} < \infty$ is used in Gretton* et al. *(2009), which is a special case of this lemma.*

Lemma 2 *If there exist $\theta_0 > 0$ and $\tilde{\theta}_0 > 0$ subject to Assumption 2, Assumption 2 is true for any $\theta \geqslant \theta_0, \tilde{\theta} \geqslant \tilde{\theta}_0$.*

Given Assumptions and Lemmas above, we have the following Theorem 1.

Theorem 1 *Suppose both Assumptions 1 and 2 hold, and assume $\frac{\partial k^v(\boldsymbol{x}_1, \boldsymbol{x}_2)}{\partial^v \boldsymbol{x}_1}$ and $\frac{\partial \tilde{k}^{\tilde{v}}(\boldsymbol{y}_1, \boldsymbol{y}_2)}{\partial^{\tilde{v}} \boldsymbol{y}_1}$ exist and are continuous for certain v and $\tilde{v}$. If there exist $\theta_0, \tilde{\theta}_0 > 0$ satisfying conditions that $\theta_0(v + \frac{1}{2}) > 1$ and $\tilde{\theta}_0(\tilde{v} + \frac{1}{2}) > 1$. Then under H_0,*
(1)

$$wKAT(\theta, \tilde{\theta}) \xrightarrow{d} T^*_{\theta\tilde{\theta}} = \sum_{i,j=1}^{\infty} (\lambda_i^*)^\theta (\tilde{\lambda}_j^*)^{\tilde{\theta}} \chi_{ij}^2, \quad \text{as } n \to \infty,$$

for $\theta \geqslant \theta_0$ and $\tilde{\theta} \geqslant \tilde{\theta}_0$ with χ_{ij}^2s being i.i.d. Chi-square variables with one degree of freedom.
(2) wKAT$(\theta, \tilde{\theta})$ has the same asymptotic distribution as

$$T_{\theta\tilde{\theta}} = \frac{1}{n^{\theta+\tilde{\theta}}} \sum_{i,j=1}^{n} \lambda_i^\theta \tilde{\lambda}_j^{\tilde{\theta}} \chi_{ij}^2,$$

for $\theta \geqslant \theta_0$ and $\tilde{\theta} \geqslant \tilde{\theta}_0$.

Proof. (1) Based on eigenvalue decomposition, we have

$$\mathbf{HSH} = \sum_{i=1}^{n} \lambda_i q_i q_i^\top, \ \mathbf{H\tilde{S}H} = \sum_{j=1}^{n} \tilde{\lambda}_j \tilde{q}_j \tilde{q}_j^\top.$$

Define $\Psi_\theta = (\psi_{1,\theta}(x), \cdots, \psi_{n,\theta}(x))$ with $\psi_{i,\theta}(x) = \lambda_i^{\theta/2} q_i$, and $\tilde{\Psi}_{\tilde{\theta}} = (\tilde{\psi}_{1,\tilde{\theta}}(y), \cdots, \tilde{\psi}_{n,\tilde{\theta}}(y))$ with $\tilde{\psi}_{j,\tilde{\theta}}(y) = \tilde{\lambda}_j^{\tilde{\theta}/2} \tilde{q}_j$. Then

$$\mathbf{HSH}^\theta = \Psi_\theta \Psi_\theta^\top = \sum_{i=1}^n \psi_{i,\theta}(x)\psi_{i,\theta}(x)^\top,\ \mathbf{H\tilde{S}H}^{\tilde{\theta}} = \tilde{\Psi}_{\tilde{\theta}} \tilde{\Psi}_{\tilde{\theta}}^\top = \sum_{j=1}^n \tilde{\psi}_{j,\tilde{\theta}}(y)\tilde{\psi}_{j,\tilde{\theta}}(y)^\top.$$

Let

$$S_{ij,\theta\tilde{\theta}} = \frac{1}{n^{(\theta+\tilde{\theta}-1)/2}} \psi_{i,\theta}(x)^\top \tilde{\psi}_{j,\tilde{\theta}}(y) = \frac{1}{n^{(\theta+\tilde{\theta}-1)/2}} \sum_{t=1}^n \psi_{i,\theta}(x_t)\tilde{\psi}_{j,\tilde{\theta}}(y_t),$$

where $\psi_{i,\theta}(x_t)$ is the t-th elements of $\psi_{i,\theta}(x)$ and $\tilde{\psi}_{j,\tilde{\theta}}(y_t)$ has the same meaning. So

$$\text{wKAT}(\theta, \tilde{\theta}) = \frac{1}{n^{\theta+\tilde{\theta}-1}} \text{tr}(\Psi_\theta \Psi_\theta^\top \tilde{\Psi}_{\tilde{\theta}} \tilde{\Psi}_{\tilde{\theta}}^\top) = \frac{1}{n^{\theta+\tilde{\theta}-1}} \text{tr}(\Psi_\theta^\top \tilde{\Psi}_{\tilde{\theta}} \tilde{\Psi}_{\tilde{\theta}}^\top \Psi_\theta) = \sum_{i,j=1}^n S_{ij,\theta\tilde{\theta}}^2$$

Based on the proof of Theorem 3 in Zhang *et al.* (2012), under H_0, and for any r such that $\lambda_{r+1}^* \neq \lambda_r^*$, $\tilde{\lambda}_{r+1}^* \neq \tilde{\lambda}_r^*$, we have

$$\sum_{i,j=1}^r S_{ij,\theta\tilde{\theta}}^2 \xrightarrow{d} \sum_{i,j=1}^r (\lambda_i^*)^\theta (\tilde{\lambda}_j^*)^{\tilde{\theta}} \chi_{ij}^2 \triangleq T_{r,\theta\tilde{\theta}}^*, \quad \text{as } n \to \infty,$$

where χ_{ij}^2s are *i.i.d.* Chi-squared random variables with one degree of freedom.

Next we continue to prove that this result also holds when $r = n \to \infty$. According to Lemma 1 and Lemma 2, $\sum_{i=1}^\infty (\lambda_i^*)^\theta < \infty$ and $\sum_{i=1}^\infty (\tilde{\lambda}_j^*)^{\tilde{\theta}} < \infty$ for any $\theta \geqslant \theta_0$ and $\tilde{\theta} \geqslant \tilde{\theta}_0$. It follows that $E(T_{\theta\tilde{\theta}}^*) = \sum_{i,j=1}^\infty (\lambda_i^*)^\theta (\tilde{\lambda}_j^*)^{\tilde{\theta}} < \infty$ and $\text{var}(T_{\theta\tilde{\theta}}^*) = 2\sum_{i,j=1}^\infty (\lambda_i^*)^{2\theta} (\tilde{\lambda}_j^*)^{2\tilde{\theta}} < \infty$. Thus $T_{\theta\tilde{\theta}}^*$ is bounded in probability by Chebyshev's inequality, which implies that $\sum_{i,j=1}^\infty S_{ij,\theta\tilde{\theta}}^2$ is bounded in probability as $n \to \infty$. For each $\epsilon > 0$,

$$\lim_{n,r\to\infty} \limsup P(|\sum_{i,j=1}^r S_{ij,\theta\tilde{\theta}}^2 - \sum_{i,j=1}^n S_{ij,\theta\tilde{\theta}}^2| \geqslant \epsilon) \leqslant \lim_{R_0\to\infty} P(\sum_{i,j=R_0}^\infty S_{ij,\theta\tilde{\theta}}^2 \geqslant \epsilon) = 0,$$

where $R_0 = \min\{n, r\}$. Using the Lemma 9 of Zhang *et al.* (2012), we can conclude that $\text{wKAT}(\theta, \tilde{\theta})$ converge in distribution to $T_{\theta\tilde{\theta}}^*$ as $n \to \infty$.

(2) By Lemma 2, we have $\sum_{i=1}^\infty \left|(\lambda_i/n)^\theta - (\lambda_i^*)^\theta\right| \xrightarrow{p} 0$ and $\sum_{j=1}^\infty \left|(\tilde{\lambda}_j/n)^{\tilde{\theta}} - (\tilde{\lambda}_j^*)^{\tilde{\theta}}\right| \xrightarrow{p} 0$ for any $\theta \geqslant \theta_0$ and $\tilde{\theta} \geqslant \tilde{\theta}_0$. Then it follows that

$$\begin{aligned}
&\sum_{i,j=1}^\infty \left|(\frac{\lambda_i}{n})^\theta (\frac{\tilde{\lambda}_j}{n})^{\tilde{\theta}} - (\lambda_i^*)^\theta (\tilde{\lambda}_j^*)^{\tilde{\theta}}\right| \\
&\leqslant \sum_{i=1}^\infty (\lambda_i^*)^\theta \sum_{j=1}^\infty \left|(\frac{\tilde{\lambda}_j}{n})^{\tilde{\theta}} - (\tilde{\lambda}_j^*)^{\tilde{\theta}}\right| + \sum_{j=1}^\infty (\tilde{\lambda}_j^*)^{\tilde{\theta}} \sum_{i=1}^\infty \left|(\frac{\lambda_i}{n})^\theta - (\lambda_i^*)^\theta\right| \qquad (1) \\
&\quad + \sum_{i=1}^\infty \left|(\frac{\lambda_i}{n})^\theta - (\lambda_i^*)^\theta\right| \sum_{j=1}^\infty \left|(\frac{\tilde{\lambda}_j}{n})^{\tilde{\theta}} - (\tilde{\lambda}_j^*)^{\tilde{\theta}}\right| \xrightarrow{p} 0, \quad \text{as } n \to \infty.
\end{aligned}$$

Denote $\lambda_{ij,\theta\tilde{\theta}} = \lambda_i^{\theta}\tilde{\lambda}_j^{\tilde{\theta}}/n^{\theta+\tilde{\theta}}$ and $\lambda^*_{ij,\theta\tilde{\theta}} = (\lambda^*_i)^{\theta}(\tilde{\lambda}^*_j)^{\tilde{\theta}}$. Following the proof of Theorem 1 in Gretton *et al.* (2009) and the Cauchy-Schwarz inequality, we can obtain that as $n \to \infty$,

$$
\begin{aligned}
&|\sum_{i,j=1}^{\infty}(\lambda_{ij,\theta\tilde{\theta}} - \lambda^*_{ij,\theta\tilde{\theta}})\chi^2_{ij}| \\
\leqslant &|\sum_{i,j=1}^{\infty}\lambda^{1/2}_{ij,\theta\tilde{\theta}}\big(\lambda^{1/2}_{ij,\theta\tilde{\theta}} - (\lambda^*_{ij,\theta\tilde{\theta}})^{1/2}\big)\chi^2_{ij}| + |\sum_{i,j=1}^{\infty}\big(\lambda^{1/2}_{ij,\theta\tilde{\theta}} - (\lambda^*_{ij,\theta\tilde{\theta}})^{1/2}\big)(\lambda^*_{ij,\theta\tilde{\theta}})^{1/2}\chi^2_{ij}| \\
\leqslant &\{\sum_{i,j=1}^{\infty}\lambda_{ij,\theta\tilde{\theta}}{\chi^2_{ij}}^2\}^{1/2}\{\sum_{i,j=1}^{\infty}|\lambda^{1/2}_{ij,\theta\tilde{\theta}} - (\lambda^*_{ij,\theta\tilde{\theta}})^{1/2}|^2\}^{1/2} + \{\sum_{i,j=1}^{\infty}\lambda^*_{ij,\theta\tilde{\theta}}{\chi^2_{ij}}^2\}^{1/2}\{\sum_{i,j=1}^{\infty}|\lambda^{1/2}_{ij,\theta\tilde{\theta}} - (\lambda^*_{ij,\theta\tilde{\theta}})^{1/2}|^2\}^{1/2} \\
\leqslant &\{\sum_{i,j=1}^{\infty}\lambda_{ij,\theta\tilde{\theta}}{\chi^2_{ij}}^2\}^{1/2}\{\sum_{i,j=1}^{\infty}|\lambda_{ij,\theta\tilde{\theta}} - \lambda^*_{ij,\theta\tilde{\theta}}|\}^{1/2} + \{\sum_{i,j=1}^{\infty}\lambda^*_{ij,\theta\tilde{\theta}}{\chi^2_{ij}}^2\}^{1/2}\{\sum_{i,j=1}^{\infty}|\lambda_{ij,\theta\tilde{\theta}} - \lambda^*_{ij,\theta\tilde{\theta}}|\}^{1/2} \xrightarrow{p} 0,
\end{aligned}
$$

where $\sum_{i,j=1}^{\infty}\lambda_{ij,\theta\tilde{\theta}}{\chi^2_{ij}}^2$ and $\sum_{i,j=1}^{\infty}\lambda^*_{ij,\theta\tilde{\theta}}{\chi^2_{ij}}^2$ are bounded in probability by Chebyshev's inequality and $\sum_{i,j=1}^{\infty}|\lambda_{ij,\theta\tilde{\theta}} - \lambda^*_{ij,\theta\tilde{\theta}}|$ converges to 0 in probability by (1). Consequently, wKAT$(\theta,\tilde{\theta})$ and $T_{\theta\tilde{\theta}}$ have the same asymptotic distribution. This completes the proof of Theorem 1.

Based on Theorem 1, the asymptotic mean $\mu_{\theta\tilde{\theta}}$ and variance $D^2_{\theta\tilde{\theta}}$ of wKAT$(\theta,\tilde{\theta})$ can be estimated via $T_{\theta\tilde{\theta}}$ as follows:

$$
\hat{\mu}_{\theta\tilde{\theta}} = E(T_{\theta\tilde{\theta}}) = \frac{1}{n^{\theta+\tilde{\theta}}}\sum_{i,j=1}^{n}\lambda_i^{\theta}\tilde{\lambda}_j^{\tilde{\theta}},
$$

$$
\hat{D}^2_{\theta\tilde{\theta}} = \mathrm{Var}(T_{\theta\tilde{\theta}}) = \frac{2}{n^{2(\theta+\tilde{\theta})}}\sum_{i,j=1}^{n}\lambda_i^{2\theta}\tilde{\lambda}_j^{2\tilde{\theta}}.
$$

Denote $T^*_{n,\theta\tilde{\theta}} = \sum_{i,j=1}^{n}(\lambda^*_i)^{\theta}(\tilde{\lambda}^*_j)^{\tilde{\theta}}\chi^2_{ij}$ and its moments $\mu_{n,\theta\tilde{\theta}} = E(T^*_{n,\theta\tilde{\theta}}) = \sum_{i,j=1}^{n}(\lambda^*_i)^{\theta}(\tilde{\lambda}^*_j)^{\tilde{\theta}}$, $D^2_{n,\theta\tilde{\theta}} = \mathrm{Var}(T^*_{n,\theta\tilde{\theta}}) = 2\sum_{i,j=1}^{n}(\lambda^*_i)^{2\theta}(\tilde{\lambda}^*_j)^{2\tilde{\theta}}, \theta \in \Theta, \tilde{\theta} \in \tilde{\Theta}$. The following Theorem 2 shows the asymptotic distribution of $T^*_{n,\theta\tilde{\theta}}$ on $(\theta,\tilde{\theta}) \in \Theta \times \tilde{\Theta}$, the Cartesian product of sets Θ and $\tilde{\Theta}$.

Theorem 2 *Assume that for any subset $\Omega \subset \Theta \times \tilde{\Theta}$ and constants $u_{\theta\tilde{\theta}} < \infty, (\theta,\tilde{\theta}) \in \Theta \times \tilde{\Theta}$, the following statement holds,*

$$
\lim_{n\to\infty}\frac{\sum_{i,j=1}^{n}\big(\sum_{(\theta,\tilde{\theta})\in\Omega}u_{\theta\tilde{\theta}}(\lambda^*_i)^{\theta}(\tilde{\lambda}^*_j)^{\tilde{\theta}}\big)^4}{\big[\sum_{i,j=1}^{n}\big(\sum_{(\theta,\tilde{\theta})\in\Omega}u_{\theta\tilde{\theta}}(\lambda^*_i)^{\theta}(\tilde{\lambda}^*_j)^{\tilde{\theta}}\big)^2\big]^2} = 0.
$$

Then $\big(\frac{T^*_{n,\theta\tilde{\theta}} - \mu_{n,\theta\tilde{\theta}}}{D_{n,\theta\tilde{\theta}}}\big)_{(\theta,\tilde{\theta})\in\Theta\times\tilde{\Theta}} \to \mathbb{X}_2$ as $n \to \infty$, and $\mathbb{X}_2$ is a two dimensional Gaussian random field.

Proof. When Ω has only one element $(\theta, \tilde{\theta})$ and $u_{\theta\tilde{\theta}} = 1$, the assumption in Theorem 2 indicates that the Lyapunov condition is satisfied. So $\frac{T^*_{n,\theta\tilde{\theta}} - \mu_{n,\theta\tilde{\theta}}}{D_{n,\theta\tilde{\theta}}}$ asymptotically follows the standard normal distribution.

Next, we are to establish the asymptotic normality of the multivariate $\big(\frac{T^*_{n,\theta\tilde{\theta}} - \mu_{n,\theta\tilde{\theta}}}{D_{n,\theta\tilde{\theta}}}\big)_{(\theta,\tilde{\theta})\in\Omega}$ for any $\Omega \subset \Theta \times \tilde{\Theta}$ with more than one elements. To prove this, we need to induce the asymptotic normality for its any linear combination. Given any coefficient set $\{u_{\theta\tilde{\theta}}, (\theta, \tilde{\theta}) \in \Theta \times \tilde{\Theta}\}$, its linear combination is $\sum_{i,j=1}^{n} (\sum_{(\theta,\tilde{\theta})\in\Omega} u_{\theta\tilde{\theta}} (\lambda^*_i)^{\theta} (\tilde{\lambda}^*_j)^{\tilde{\theta}}) \chi^2_{ij}$. And it is asymptotic normal when the Lyapunov condition holds, that is when $\frac{\sum_{i,j=1}^{n} \big(\sum_{(\theta,\tilde{\theta})\in\Omega} u_{\theta\tilde{\theta}} (\lambda^*_i)^{\theta} (\tilde{\lambda}^*_j)^{\tilde{\theta}}\big)^4}{\big[\sum_{i,j=1}^{n} \big(\sum_{(\theta,\tilde{\theta})\in\Omega} u_{\theta\tilde{\theta}} (\lambda^*_i)^{\theta} (\tilde{\lambda}^*_j)^{\tilde{\theta}}\big)^2\big]^2}$ approaches zero when n goes to infinity, which is a direct use of the assumption in Theorem 2. Now we have that the multivariate $\big(\frac{T^*_{n,\theta\tilde{\theta}} - \mu_{n,\theta\tilde{\theta}}}{D_{n,\theta\tilde{\theta}}}\big)_{(\theta,\tilde{\theta})\in\Omega}$ asymptotically follows multivariate normal distribution. That is, any finite-dimensional subvector of $\big(\frac{T^*_{n,\theta\tilde{\theta}} - \mu_{n,\theta\tilde{\theta}}}{D_{n,\theta\tilde{\theta}}}\big)_{(\theta,\tilde{\theta})\in\Theta\times\tilde{\Theta}}$ converges to the corresponding subvectors of $\mathbb{X}_2$. Condition (i) of Theorem 3.3.1 of Khoshnevisan (2002) is satisfied.

Denote $\omega_{T^*_n}(\delta) = \sup_{\substack{(\theta_1,\tilde{\theta}_1),(\theta_2,\tilde{\theta}_2)\in(0,1]^2, \\ |\theta_1-\theta_2|+|\tilde{\theta}_1-\tilde{\theta}_2|<\delta}} \big|\frac{T^*_{n,\theta_1\tilde{\theta}_1} - \mu_{n,\theta_1\tilde{\theta}_1}}{D_{n,\theta_1\tilde{\theta}_1}} - \frac{T^*_{n,\theta_2\tilde{\theta}_2} - \mu_{n,\theta_2\tilde{\theta}_2}}{D_{n,\theta_2\tilde{\theta}_2}}\big|$. Based on continuity of exponential function, for all $\epsilon > 0$, $\lim_{\delta\to 0} \limsup_{n\to\infty} \mathrm{P}(\omega_{T^*_n}(\delta) \geq \epsilon) = 0$. Condition (ii) of Theorem 3.3.1 of Khoshnevisan (2002) is satisfied. So following this Theorem, we have that $\big(\frac{T^*_{n,\theta\tilde{\theta}} - \mu_{n,\theta\tilde{\theta}}}{D_{n,\theta\tilde{\theta}}}\big)_{(\theta,\tilde{\theta})\in\Theta\times\tilde{\Theta}} \to \mathbb{X}_2$, and $\mathbb{X}_2$ is a gaussian random field.

Remark 3 *We point out that the assumption in Theorem 2 is mild. For example, when there exists no eigenvalue dominating the rest ones, the assumption holds.*

3 Supplemental figures

3.1 Type I error rates for numerical simulations

Figure S1. Barplots for the type I error rates of KAT, MaxKAT.perm and MaxKAT.gev under Model II when Δ_y is of autoregressive structure.
Figure S2. Barplots for the type I error rates of KAT, MaxKAT.perm and MaxKAT.gev under Model II when Δ_y is of compound-symmetry structure.
Figure S3. Barplots for the type I error rates of KAT, MaxKAT.perm and MaxKAT.gev under Model III when Δ_y is of autoregressive structure.
Figure S4. Barplots for the type I error rates of KAT, MaxKAT.perm and MaxKAT.gev under Model III when Δ_y is of compound-symmetry structure.

3.2 Empirical powers for numerical simulations

Figure S5. The empirical powers of KAT, MaxKAT.perm and MaxKAT.gev under Model II when Δ_y is of autoregressive structure.

Figure S6. The empirical powers of KAT, MaxKAT.perm and MaxKAT.gev under Model II when Δ_y is of compound-symmetry structure.

Figure S7. The empirical powers of KAT, MaxKAT.perm and MaxKAT.gev under Model III when Δ_y is of autoregressive structure.

Figure S8. The empirical powers of KAT, MaxKAT.perm and MaxKAT.gev under Model III when Δ_y is of compound-symmetry structure.

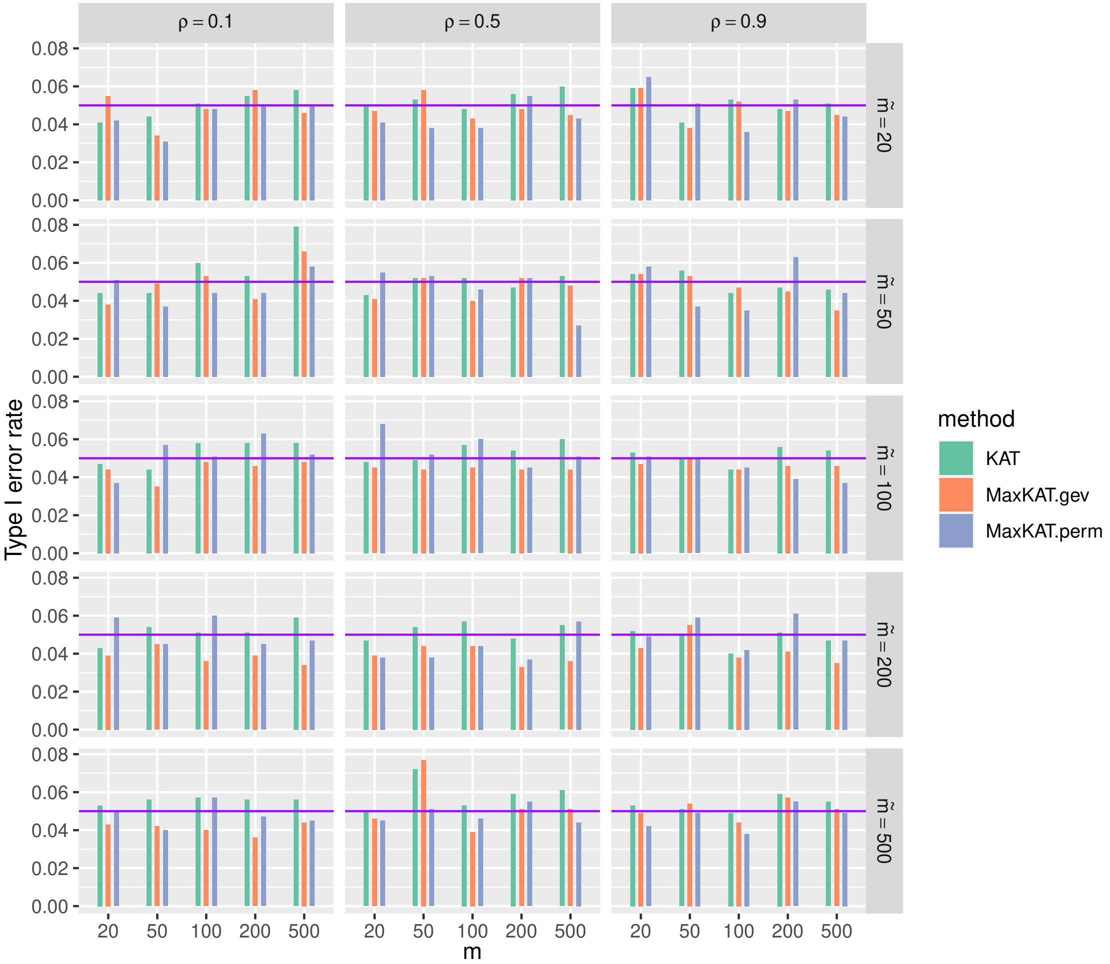

Figure S1: Barplots for the type I error rates of KAT, MaxKAT.perm and MaxKAT.gev under Model II when Δ_y is of autoregressive structure.

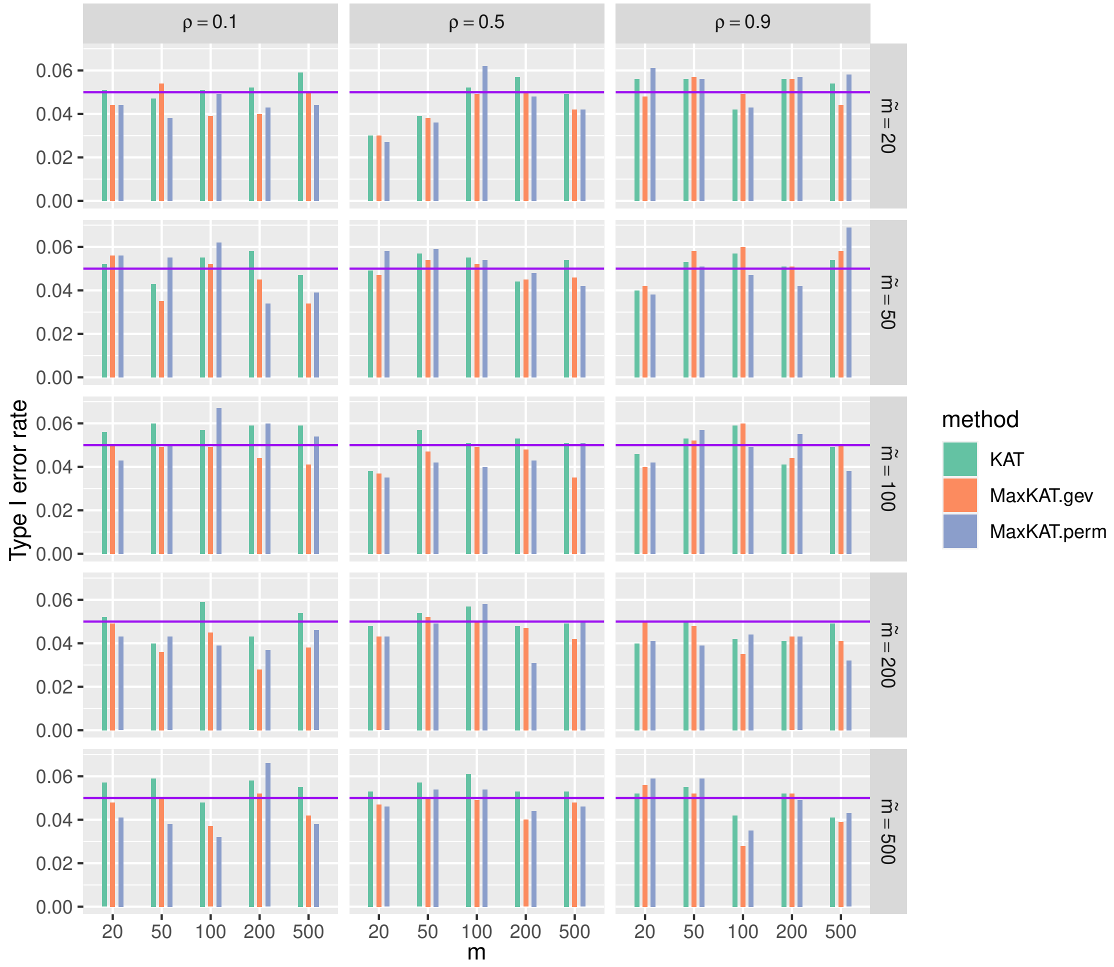

Figure S2: Barplots for the type I error rates of KAT, MaxKAT.perm and MaxKAT.gev under Model II when Δ_y is of compound-symmetry structure.

References

Gretton, A., Fukumizu, K., Harchaoui, Z. and Sriperumbudur, B.(2009). A fast, consistent kernel two-sample test. *Advances in Neural Information Processing Systems* **23**, 673–681.

Khoshnevisan, D. (2002). *Multiparameter Processes : An Introduction to Random Fields.* Springer-Verlag New York, Inc.

Weyl, H. (1912). Das asymptotische verteilungsgesetz der eigenwerte linearer partieller dif-

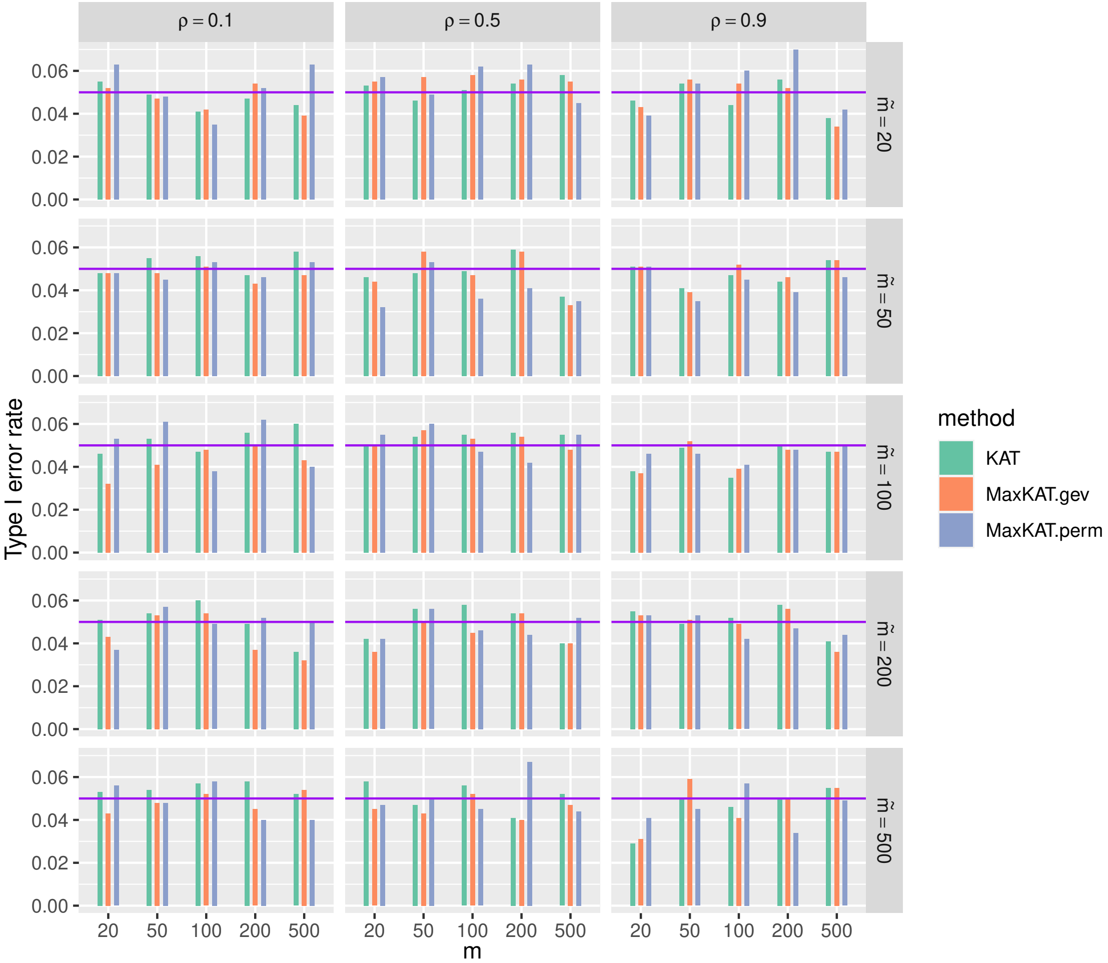

Figure S3: Barplots for the type I error rates of KAT, MaxKAT.perm and MaxKAT.gev under Model III when Δ_y is of autoregressive structure.

ferentialgleichungen (mit einer anwendung auf die theorie der hohlraumstrahlung). *Mathematische Annalen* **71**, 441–479.

Zhang, K., Peters, J., Janzing, D. and Schölkopf, B. (2012). Kernel-based conditional independence test and application in causal discovery. *In Proceedings of the Conference on Uncertainty in Artificial Intelligence (UAI)*, 804-813.

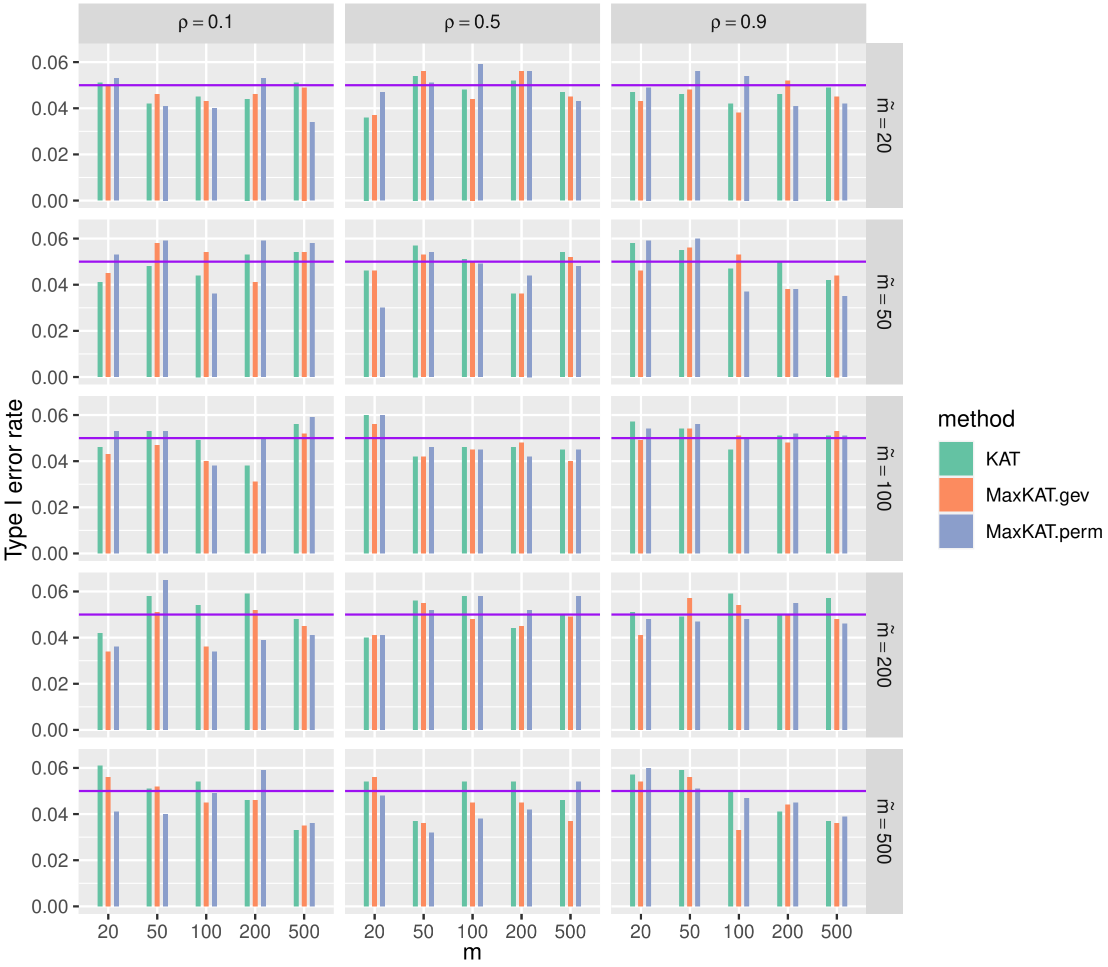

Figure S4: Barplots for the type I error rates of KAT, MaxKAT.perm and MaxKAT.gev under Model III when Δ_y is of compound-symmetry structure.

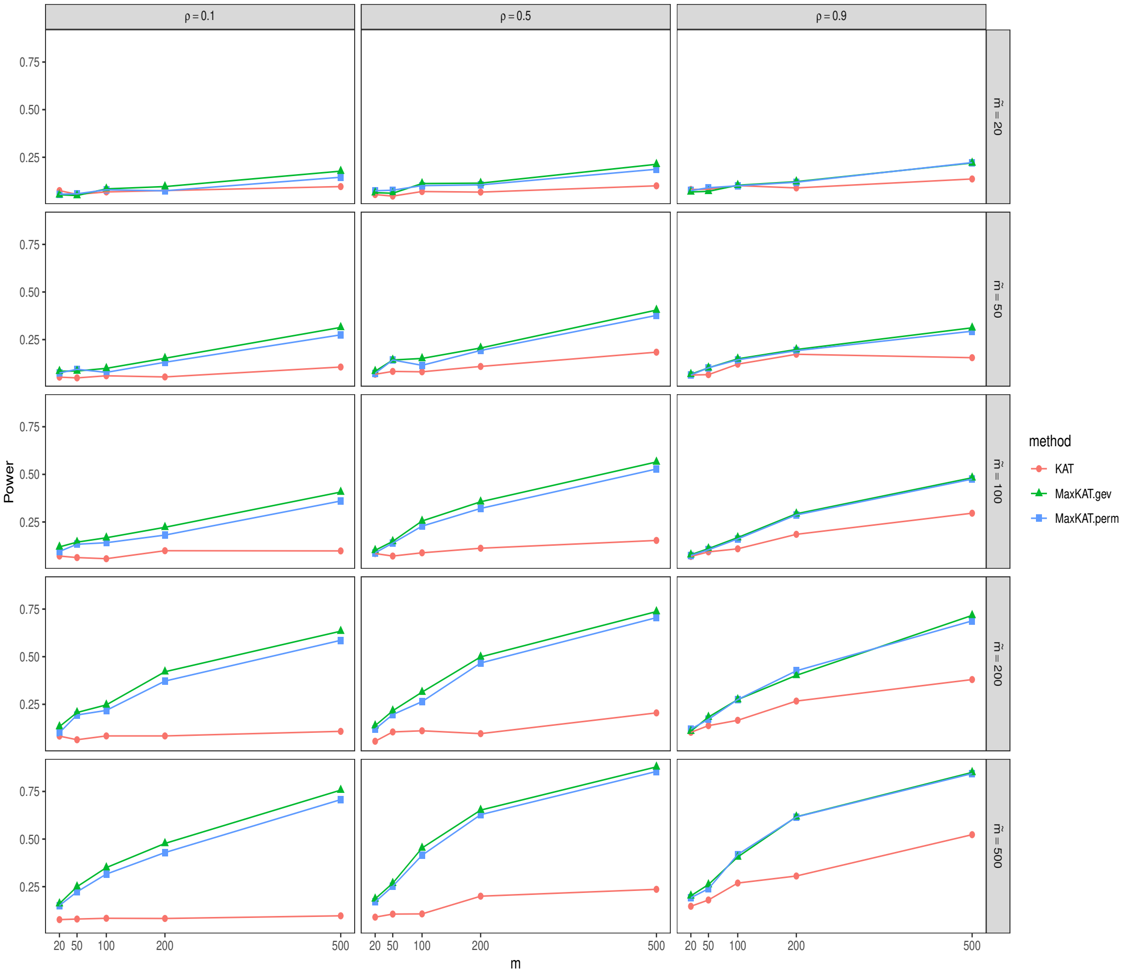

Figure S5: The empirical powers of KAT, MaxKAT.perm and MaxKAT.gev under Model II when Δ_y is of autoregressive structure.

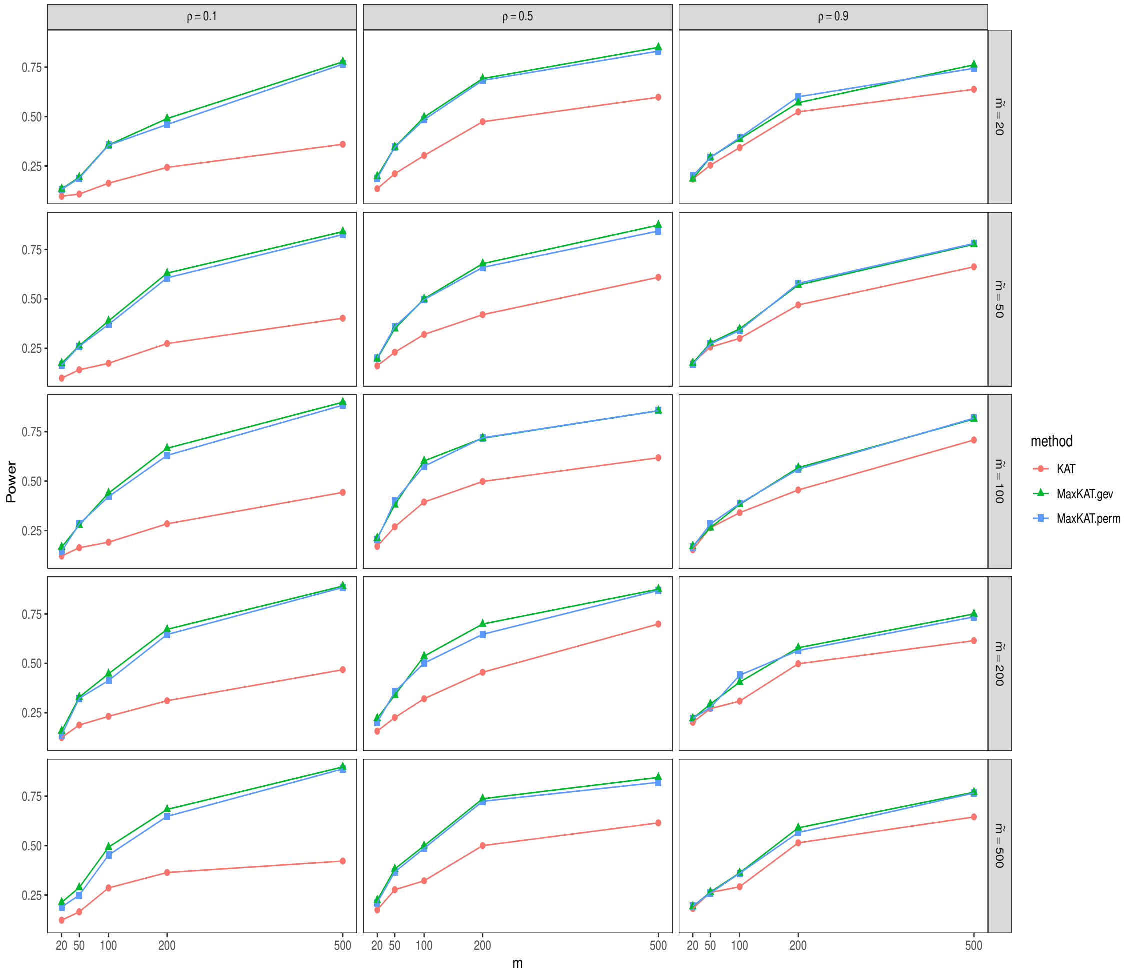

Figure S6: The empirical powers of KAT, MaxKAT.perm and MaxKAT.gev under Model II when Δ_y is of compound-symmetry structure.

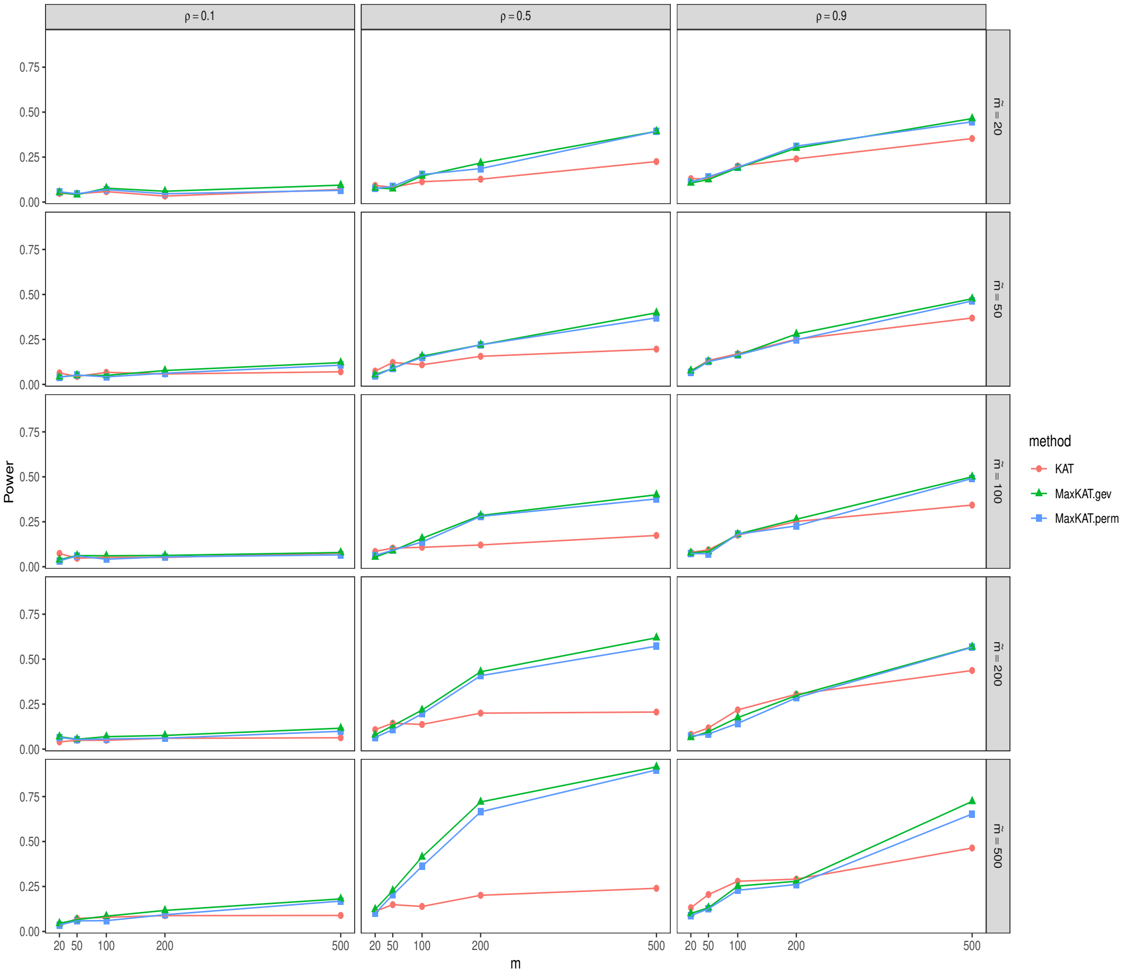

Figure S7: The empirical powers of KAT, MaxKAT.perm and MaxKAT.gev under Model III when Δ_y is of autoregressive structure.

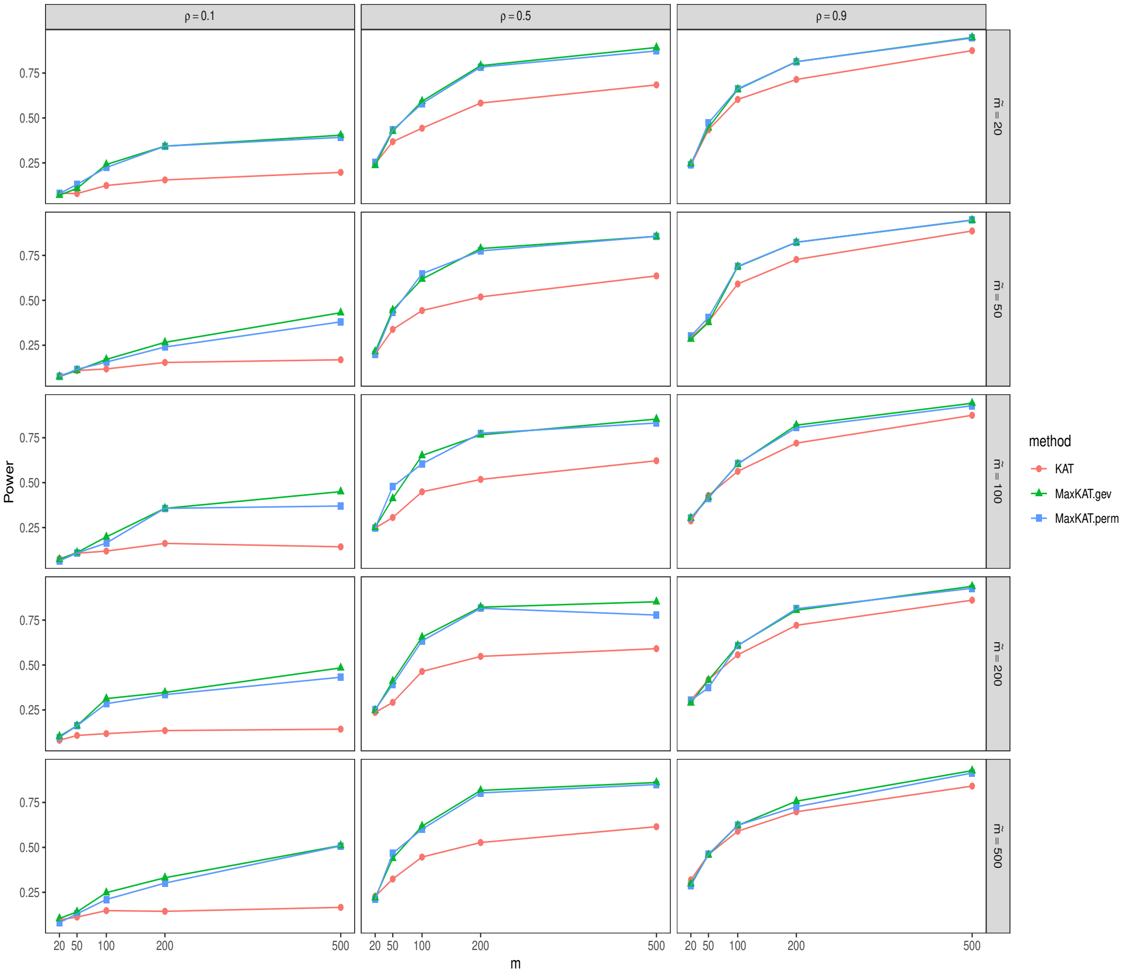

Figure S8: The empirical powers of KAT, MaxKAT.perm and MaxKAT.gev under Model III when Δ_y is of compound-symmetry structure.
